# *Varroa destructor* parasitism has a greater effect on proteome changes than the deformed wing virus and activates TGF-β signaling pathways

**DOI:** 10.1038/s41598-019-45764-1

**Published:** 2019-06-28

**Authors:** Tomas Erban, Bruno Sopko, Klara Kadlikova, Pavel Talacko, Karel Harant

**Affiliations:** 1 Crop Research Institute, Drnovska 507/73, Prague 6-Ruzyne, CZ-161 06 Czechia; 2 Department of Plant Protection, Faculty of Agrobiology, Food and Natural Resources, Czech University of Life Sciences, Prague 6-Suchdol, CZ-165 00 Czechia; 3 Proteomics Core Facility, Faculty of Science, Charles University, BIOCEV, Prumyslova 595, Vestec, CZ-25242 Czechia

**Keywords:** Parasite host response, Viral host response

## Abstract

Honeybee workers undergo metamorphosis in capped cells for approximately 13 days before adult emergence. During the same period, *Varroa* mites prick the defenseless host many times. We sought to identify proteome differences between emerging *Varroa*-parasitized and parasite-free honeybees showing the presence or absence of clinical signs of deformed wing virus (*DWV*) in the capped cells. A label-free proteomic analysis utilizing nanoLC coupled with an Orbitrap Fusion Tribrid mass spectrometer provided a quantitative comparison of 2316 protein hits. Redundancy analysis (RDA) showed that the combination of *Varroa* parasitism and *DWV* clinical signs caused proteome changes that occurred in the same direction as those of *Varroa* alone and were approximately two-fold higher. Furthermore, proteome changes associated with *DWV* signs alone were positioned above *Varroa* in the RDA. Multiple markers indicate that *Varroa* activates TGF-β-induced pathways to suppress wound healing and the immune response and that the collective action of stressors intensifies these effects. Furthermore, we indicate JAK/STAT hyperactivation, p53-BCL-6 feedback loop disruption, Wnt pathway activation, Wnt/Hippo crosstalk disruption, and NF-κB and JAK/STAT signaling conflict in the *Varroa*–honeybee–*DWV* interaction. These results illustrate the higher effect of *Varroa* than of *DWV* at the time of emergence. Markers for future research are provided.

## Introduction

Since *Varroa destructor* (Anderson & Trueman, 2000) shifted from the eastern honeybee, *Apis cerana* Fabricius, 1793, to the western honeybee, *Apis mellifera* Linnaeus, 1758, this mite has become one of the most important factors in colony loss, and *Varroa* parasitism is strongly connected to viral transmission within and between colonies. Historical evidence shows that viruses often exist in latent phases until *Varroa* parasitism occurs in honeybees^1^. Although diverse pathogenic viruses have been identified in honeybees, the most common and most well-studied is the deformed wing virus (*DWV*), for which increases in load with mite infestation have been thoroughly documented in honeybee colonies. This tight connection between mite and *DWV* occurrence nicely demonstrates the finding that *Varroa* decreases *DWV* diversity in its host^2^. Moreover, the recent global spread of *DWV* is driven by *Varroa* transmission from European to North American honeybee populations^3^.

Although *DWV* transmission by *Varroa* is certain^2–5^, the molecular mechanisms underlying the *Varroa*–honeybee–*DWV* interaction are poorly understood. *Varroa* parasitism in capped cells leads to immunosuppression in bees, likely increasing the probability of *DWV* amplification^6,7^. It has been indicated that the virus, not *Varroa*, downregulates NF-κB through the suppression of dorsal-1A^8^. Furthermore, it was indicated that *Varroa*-vectored *DWV* suppresses NF-κB activation through its negative modulator, Amel\LRR, with silenced dorsal-1A^9^. Other studies have found little evidence of immunosuppression by *Varroa* parasitism in honeybee genes^10–12^, and it has been suggested that variations in the results of functional studies on *Varroa*-exposed honeybees are possibly due to the analysis of different bee development ages^4^. Furthermore, Doublet *et al*.^13^ compared transcriptomic studies and suggested that gene expression can be influenced by experimental design.

The honeybee individual undergoes complete metamorphosis. Honeybee workers typically develop for 21 days until emergence and are sealed in the brood cells for approximately 13 days^14^. Because there can be variations in the time of development, it is useful to select samples for which the stage is easily recognizable. Useful signs can be, for example, pigmentation of the pupa eyes^15,16^ and the time when the honeybee individual emerges^16,17^. Our comparison of the hemolymph proteome between red-eye pupae and emerging worker bees showed a drastic decrease in protein abundance at the time of emergence^17^. Furthermore, pupae with differently pigmented eyes and emerging bees were used to study body weight loss due to *Varroa* parasitism in drone pupae, and body weight loss was most substantial in emerging adults^16^. Some studies were performed to show the effects of *Varroa* and/or *DWV* on gene expression in the pupa. Ryabov *et al*.^4^ performed manipulative experiments with *Varroa* and *DWV*, and transcriptome analyses of samples of purple-eye pupae (15^th^ day of development) showed changes in several genes involved in development and the immune response, which may be key for *DWV* pathogenesis^4^. Furthermore, a proteomic study attempted to show differences in *Varroa*-parasitized workers and drone purple-eye pupae^18^. However, relevant studies have not been performed in emerging bees, which are useful for exact comparative physiological studies^17^. Furthermore, the impact of the mite on the bee should be more substantial at the time of adult emergence than in the red/purple-eye pupa stage. Thus, the emerging bee represents a remarkable sample type to show the effects of the mite and virus on the honeybee.

Furthermore, it can be proposed that the principle mechanisms underlying the *Varroa*–honeybee–*DWV* interaction should be similar to those established for tick–host–pathogen interactions. Both ticks and pathogens manipulate their hosts, but their mutual impact results in both conflict and cooperation^19^. The tick and the pathogen conflict in the activation of mechanisms that limit pathogen infection^19^, which at first can seem contrary to the association between increases in viral loads and *Varroa* occurrence in honeybee colonies^2–4^. However, it is important to stress that *Varroa* is transmitted between individual bees within a colony as well as between colonies^2^. Cooperation occurs because the tick facilitates pathogen infection, but the pathogen does not affect the feeding and reproduction of the parasite^19^. However, the molecular mechanisms that provide evidence of the *Varroa*–honeybee–*DWV* interaction are puzzling compared to those in the tick–host–pathogen interaction.

**Figure 1:**
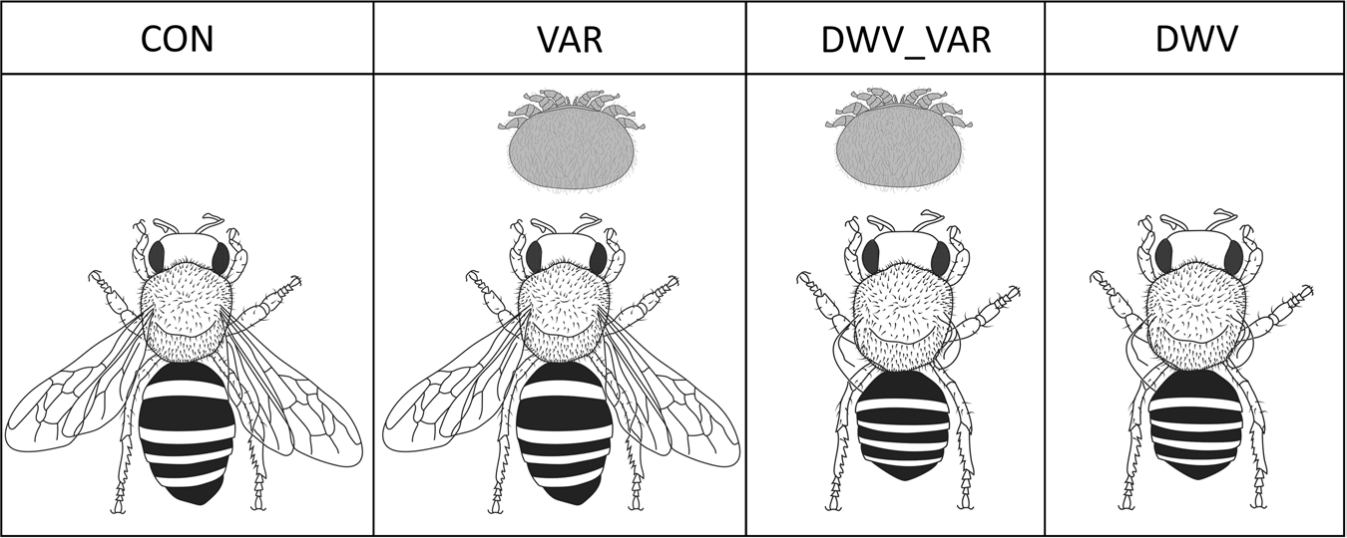
The samples consisted of emerging worker honeybees collected just as they started opening the capped brood cells. The bees were or were not parasitized by *Varroa* mites in the capped cells and did or did not possess visible clinical signs of *DWV*. Thus, the following four variants were collected from heavily *Varroa*-infested colonies: **CON** – control, nonparasitized bees without *DWV* clinical signs; **VAR** – *Varroa*-parasitized bees without visible *DWV* clinical signs; **DWV_VAR** – *Varroa*-parasitized bees with visible *DWV* clinical signs; **DWV** – *Varroa* nonparasitized bees with *DWV* clinical signs.

In this study, we aimed to show the impact of *Varroa* mite parasitization, either with or without the presence of clinical signs of *DWV*, on the proteome of worker bees at the time when they emerge. Thus, we analyzed worker bees collected just as they were emerging/started opening the cell cap^17^. We designed four variants of bee samples (Fig. 1) that were analyzed by a high-throughput label-free proteomic approach in which we utilized the power of nanoliquid chromatography coupled with a state-of-the-art Orbitrap Fusion Tribrid mass spectrometer (nLC-MS/MS). We show synergic stressor effects, unravel previously unknown host–parasite interactions, and provide novel disease markers. Furthermore, we indicate the similarities in conflict and cooperation with those in the tick–host–pathogen interaction^19^. This study provides the basis for understanding the effects of *Varroa* parasitism, including *DWV*, over metamorphosis in capped cells at the molecular level.

## Results and Discussion

### Overall data evaluation

After filtering the overall quantitative proteomic data, a total of 2316 protein hits (see Table S1a–e; the proteins are arranged according to quantitative differences between treatments) were included for further evaluation. We worked with an approximately two-fold higher number of protein array compared to a proteomic study by Surlis *et al*.^18^, who identified 1195 proteins in purple-eye drone and worker pupae. Thus, we were able explore proteomic changes in great detail from the 38 consecutively performed nLC-MS/MS runs. It is important to realize that the results of this study provide comparison on the differences of proteomes among the four variants at the time of emergence. Because we counted the mites in each honeybee, we tested whether the number of mites in our experiment could be a significant factor affecting proteome changes; however, the multiple comparison p-value adjusted by Hochberg’s method^20^ (all p-values > 0.5) did not reveal significant differences among samples with different mite numbers.

### The *Varroa* effect on the proteome is in the same direction and stronger in deformed bees than in bees without *DWV* signs

**Figure 2:**
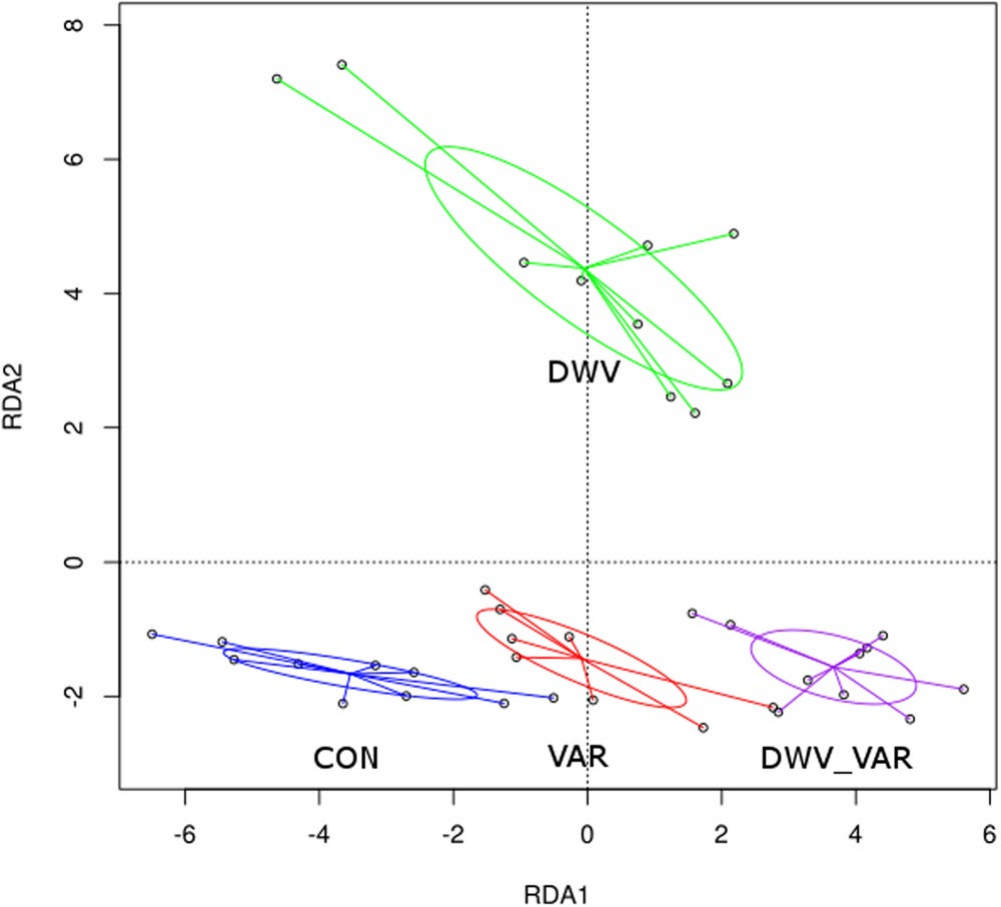
Redundancy analysis (RDA) of the proteome data showing differences caused by *Varroa* parasitism alone (VAR), deformed wing virus (DWV) signs, and the interaction of these factors (DWV_VAR) relative to control (CON) bees. The chart shows clear differences among the total proteomes of the four variants. The positioning of CON, VAR and DWV_VAR in the same direction, with the DWV variant located apart from them, indicates that *Varroa* had a higher effect on proteome changes than that with signs of the virus. Furthermore, it appears that the combination *Varroa* parasitism with *DWV* causing clinical signs is additive, as the shift of DWV_VAR away from CON is approximately two-fold greater than the shift of VAR alone. Each individual dot represents an nLC-MS/MS analysis result. RDA1 explains 65.83% of the variability, while RDA2 explains 23.83% of the variability. The results of this analysis somewhat correspond to the heatmap in Fig. 3.

**Figure 3:**
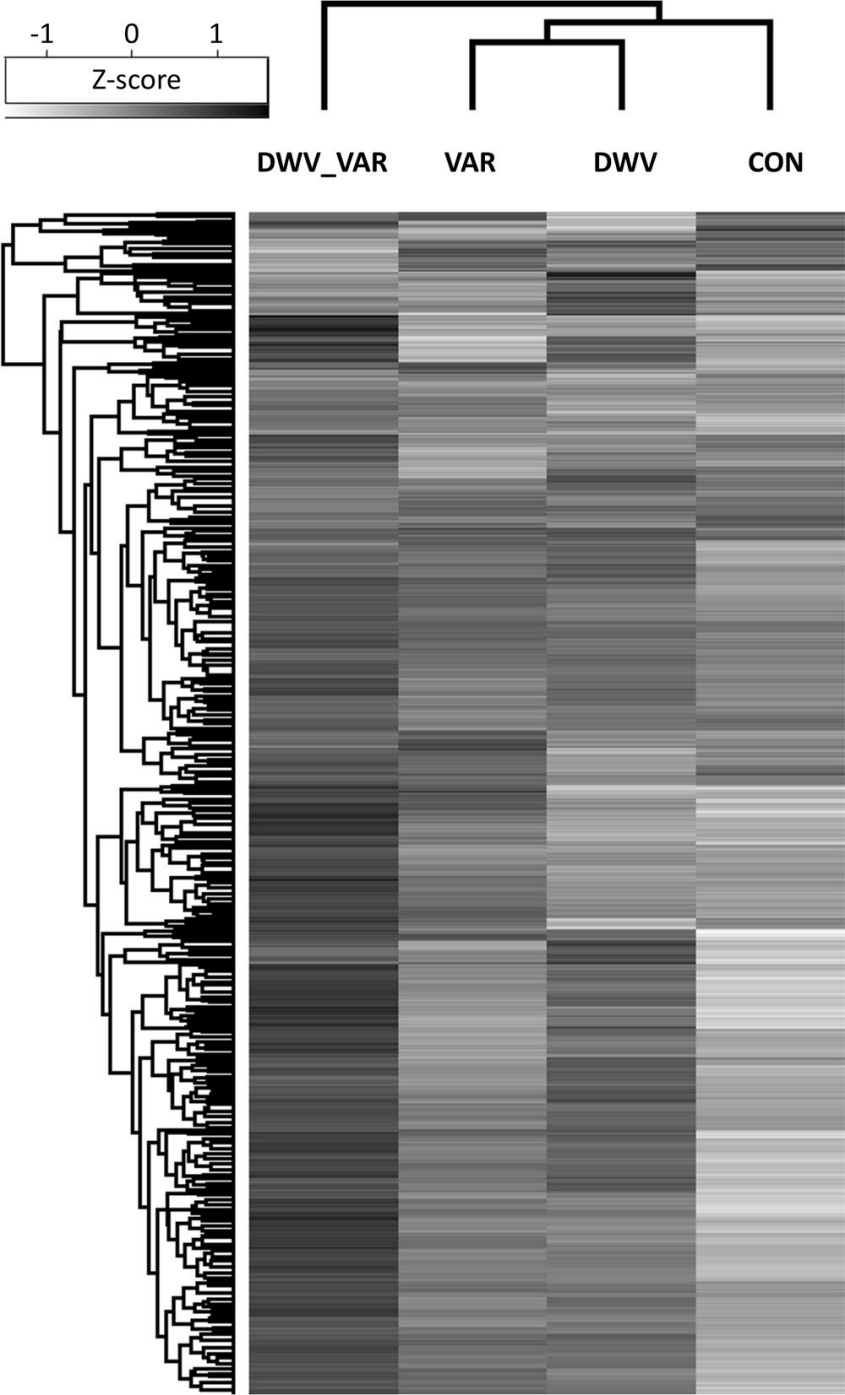
Heatmap that visualizes the proteome differences of the four variants at the time of honeybee emergence. The presentation clearly demonstrates that the proteome changes in DWV_VAR were more different than in DWV and VAR compared to CON. Thus, it appears that combination of *Varroa* parasitism and *DWV* clinical signs impact the proteome more substantially than *Varroa* and *DWV* clinical signs alone. Note that the difference between VAR and DWV variants is not visible in the hierarchical heatmap clustering in the columns; however, the difference between VAR and DWV characterizes the shift along the RDA2 axis (see Fig. 2). This heatmap was created in the Perseus environment and we also show a heatmap created in R computed from the RDA analysis (Table S1zl). Importantly, both heatmaps showed same hierarchical clustering in columns.

A redundancy analysis (RDA) (Fig. 2) as well as heatmap (Fig. 3) demonstrated clear differences among the total proteomes of the four variants. The RDA1, RDA2, and RDA3 factor scores obtained from the RDA analysis explained 65.83, 21.83, and 12.34% (Fig. S1), respectively, of the variability in protein abundance of the measured proteomes. The p-values of the multiple comparison were p = 0.001 for the effect of *Varroa* and *DWV* and p = 0.025 for the interaction of *Varroa* and *DWV*. Again, we stress that the effects observed were at the time when the worker bee emerged.

In the RDA biplot (Fig. 2), the proteomic results of the four investigated variants were distributed in four separate areas. There was a clearly visible trend for the CON, VAR, and DWV_VAR sample results, which showed a symmetrical distribution along the RDA1 axis characterizing the influence of *Varroa*; remarkably, there was no apparent shift in CON, VAR, and DWV_VAR along the RDA2 axis. Moreover, according to the RDA biplot (Fig. 2), DWV_VAR shifted from CON in the same direction as VAR by an increment of approximately 2-fold. A different situation was observed for DWV samples lacking the influence of *Varroa* in capped cells, which were positioned in the RDA chart separately above VAR. The different positions of DWV and DWV_VAR variants in the RDA biplot indicated different effects of the mite and virus on the proteome, although the analyzed bees were visually similar.

The hierarchical clustering in the columns of the heatmap (Fig. 3) clearly supports the results of RDA; however, the stronger effect of *Varroa* than *DWV* on proteome changes is visible from RDA biplot (Fig. 2). The heatmap presentation somewhat corresponds to the changes characterized by RDA1 axis alone. Thus, in the RDA differently from heatmap we can see the difference between VAR and DWV variant, which is the shift along RDA2 axis.

Thus, the results of the RDA analysis indicate that the mite has a stronger effect on proteome changes than *DWV* signs. Furthermore, it appears that the effect of mite parasitization is additive when *Varroa* parasitizes on the deformed bees. The next investigation of key markers (Table 1) indicates the unique effects of *DWV*, *Varroa* and their interaction.

**Table 1:** Selected and discussed markers.

DWV - CON	VAR - CON	DWV_VAR - CON	DWV_VAR - DWV	DWV_VAR - VAR	STRING gene/connection	Fasta headers
1.60	0.53	2.10	0.50	1.57	Clic	>XP_392027.1 chloride intracellular channel exc-4
2.00	1.08	2.67	0.66	1.59	LOC726187	>XP_006561243.1 AP-1 complex subunit beta-1
1.40	0.96	1.27	−0.13	0.31	LOC408391	>XP_003249906.1 AP-1 complex subunit mu-1
1.02	0.83	−2.65	−3.67	−3.48	Pla2/No	>CAA34681.1 phospholipase A-2
2.50	2.35	3.23	0.72	0.87	LOC724838	>XP_016769706.1 15-hydroxyprostaglandin dehydrogenase [NAD^+^]
1.51	−0.30	2.74	1.23	3.04	LOC409404	>XP_003251152.1 aminoacyl tRNA synthase complex-interacting multifunctional protein 2
0.84	0.70	2.03	1.19	1.34	LOC552324/No	>XP_624702.2 ubiquitin carboxyl-terminal hydrolase 5
1.39	0.79	2.30	0.90	1.50	LOC409521/No	>XP_393027.2 hsp70-binding protein 1
2.18	0.91	4.08	1.89	3.16	LOC724367/No	>XP_001120006.2 protein lethal(2)essential for life-like
1.43	0.86	1.93	0.50	1.07	No/No	>XP_006567844.1 PRKC apoptosis WT1 regulator protein-like
2.07	1.56	2.39	0.32	0.83	CkIIalpha	>XP_006570475.1 casein kinase II subunit alpha
−0.09	0.28	3.46	3.55	3.19	LOC724785	>XP_006564612.1 ran-binding protein 3
3.48	3.79	4.80	1.32	1.01	GB18923	>XP_397181.1 signal transducer and activator of transcription 5B
1.31	1.16	4.61	3.30	3.45	No/No	>XP_003249944.1 tetra-peptide repeat homeobox protein 1-like
0.60	0.71	2.06	1.46	1.36	No/No	>XP_006562720.1 prosaposin
0.12	0.14	1.31	1.19	1.17	raps	>XP_393796.3 G-protein-signaling modulator 2
0.66	0.26	1.06	0.40	0.80	LOC409529	>XP_393035.1 ras-like protein 2
0.62	0.48	1.04	0.42	0.56	rin	>XP_623996.3 ras GTPase-activating protein-binding protein 2
1.15	1.29	2.08	0.93	0.79	No/No	>XP_001120025.1 ras-related protein Rab-7a
−0.02	0.46	0.89	0.91	0.43	GB12838-PA	>XP_006570355.1 ras-related protein Ral-a
0.39	1.04	1.21	0.82	0.17	GB19166-PA	>XP_006564273.1 ras-related protein Rab-1A
0.56	0.69	1.12	0.56	0.43	Rab14	>XP_397056.2 PREDICTED: ras-related protein Rab-14
−0.70	0.17	0.12	0.82	−0.05	ics	>XP_395612.3 ras suppressor protein 1
1.99	1.16	2.73	0.74	1.56	LOC726291	>XP_006559926.1 rabankyrin-5
0.33	0.70	1.47	1.13	0.77	No/No	>XP_001121596.2 reagulator complex protein LAMTOR1
1.08	0.99	1.79	0.72	0.81	Rac	>XP_623951.1 ras-related protein Rac1
NaN	0.64	0.79	NaN	0.16	Cdc42	>XP_394608.2 cdc42
−0.24	0.37	−1.88	−1.64	−2.25	LOC411535	>XP_016772938.1 rho GTPase-activating protein 44
1.06	0.78	1.80	0.74	1.02	LOC412080	>XP_016768964.1 coronin-1C
0.11	0.08	0.81	0.70	0.73	gek	>XP_395596.4 serine/threonine-protein kinase Genghis Khan
0.61	0.09	0.96	0.35	0.87	bsk	>XP_016766617.1 stress-activated protein kinase JNK
0.98	0.50	1.47	0.49	0.97	Pkc	>XP_016770146.1 protein kinase C
1.63	1.15	2.41	0.79	1.26	LOC408519	>XP_006563587.1 Ste20-like STK, which is also known/assigned as STK10
1.17	0.77	1.51	0.34	0.75	LOC725374	>XP_006562044.2 dual specificity mitogen-activated protein kinase kinase 3-like
0.17	1.24	0.73	0.57	−0.51	Dsor1	>XP_393416.2 dual specificity mitogen-activated protein kinase kinase dSOR1
2.54	2.07	2.97	0.43	0.90	No/No	>XP_006565922.1 host cell factor 1
3.08	2.89	3.40	0.32	0.51	HDAC1	>XP_394976.4 histone deacetylase Rpd3
0.17	0.37	1.25	1.08	0.87	cact2	>XP_394485.2 NF-kappa-B inhibitor cactus
2.07	1.42	2.64	0.57	1.21	LOC551438	>XP_623834.1 actin-related protein 1
2.65	2.21	3.50	0.85	1.30	LOC725552	>XP_006569964.1 DNA-directed RNA polymerase III subunit RPC1
0.49	0.41	0.84	0.35	0.43	mus209	>XP_001122985.1 proliferating cell nuclear antigen
0.67	−0.10	0.26	−0.41	0.36	arm	>XP_016771134.1 armadillo segment polarity protein
0.31	0.70	1.16	0.85	0.46	GB10140	>XP_395276.6 enolase-phosphatase E1
−0.52	−1.26	−1.12	−0.61	0.14	smt3	>XP_623227.1 small ubiquitin-related modifier 3
1.06	0.49	1.89	0.82	1.40	lwr	>XP_006566478.1 SUMO-conjugating enzyme UBC9-B
0.55	0.88	2.02	1.47	1.14	RANBP2	>XP_006566105.1 RANBP2-like and GRIP domain-containing protein 5/6
0.68	1.08	1.18	0.50	0.10	LOC552748/No	>XP_625126.3 histone acetyltransferase type B catalytic subunit
0.86	0.91	1.30	0.45	0.39	No/No	>XP_006564270.1 protein arginine N-methyltransferase 1
−0.48	−0.73	0.98	1.46	1.70	LOC412588	>XP_396043.3 tyrosine-protein kinase Src42A
1.45	−3.02	−2.33	−3.79	0.68	No/No	>XP_016769030.1 basement membrane-specific heparan sulfate proteoglycan core protein
1.46	1.07	1.91	0.45	0.84	TEPA	>XP_397416.3 CD109 antigen
0.02	−0.11	1.61	1.59	1.72	mats	>XP_393046.2 MOB kinase activator-like 1
−0.15	−0.08	0.11	0.25	0.19	LOC408292	>XP_391844.3 transcriptional coactivator YAP1
0.09	0.58	1.68	1.59	1.10	LOC412708/No	>XP_396161.2 calcyclin-binding protein
0.74	0.64	1.35	0.61	0.70	No/No	>XP_016773108.1 atypical protein kinase C
1.98	0.79	2.23	0.25	1.44	par-6	>XP_001120877.2 partitioning defective protein 6
2.63	1.20	2.90	0.28	1.71	LOC409904	>XP_006570379.1 uncharacterized protein LOC409904
0.93	0.62	1.39	0.47	0.77	Camkii	>XP_006560591.1 calcium/calmodulin-dependent protein kinase II
0.66	0.89	1.44	0.79	0.55	LOC413006/No	>XP_396457.1 leucine-rich repeat flightless-interacting protein 2
2.11	0.90	3.13	1.02	2.23	LOC725041	>XP_001120937.1 slit homolog 1 protein-like
0.74	0.70	1.53	0.79	0.83	PRF	>NP_001091636.1 profilin
−0.03	0.22	0.87	0.90	0.66	LOC551313	>XP_623751.1 calcineurin subunit B type 2
0.65	1.15	1.50	0.86	0.36	No/No	>XP_006557820.1 mitogen-activated protein kinase-binding protein 1
1.04	1.34	1.59	0.56	0.25	GB19066/No	>XP_006567065.1 dorsal protein
0.86	1.42	1.23	0.37	−0.19	LOC726612	>XP_006566669.1 evolutionarily conserved signaling intermediate in Toll pathway
0.22	0.15	0.45	0.23	0.31	No/No	>NP_001104234.1 hexamerin 70a precursor
−1.77	−1.57	−0.72	1.05	0.85	No/No	>NP_001011600.1 hexamerin 70b precursor
0.68	2.86	5.26	4.58	2.41	No/No	>NP_001092187.1 hexamerin 70c precursor
−0.70	2.12	4.07	4.77	1.94	No/No	>BAI82214.1 hexamerin 110
8.29	4.13	6.77	−1.52	2.64	Mrjp1/No	>Q17060.1 Major royal jelly protein 3
7.98	4.24	3.30	−4.68	−0.94	Mrjp3/No	>O18330.1 Major royal jelly protein 1
−1.73	−0.40	−2.81	−1.08	−2.41	Vg/No	>Q868N5.1 Vitellogenin
1.40	0.36	0.94	−0.46	0.58	LOC412458	>XP_395915.4 farnesol dehydrogenase-like
0.80	0.52	1.43	0.63	0.91	LOC40615	>XP_394922.3 juvenile hormone epoxide hydrolase 1
2.77	2.48	3.18	0.41	0.70	LOC408288	>XP_006566117.1 isovaleryl-CoA dehydrogenase
0.93	0.73	1.73	0.80	0.99	Trx-2	>XP_003250408.1 thioredoxin-2
1.34	1.51	2.11	0.77	0.60	cl/No	>XP_395877.1 thioredoxin domain-containing protein 17
1.55	0.77	2.17	0.62	1.40	Trxr-1	>AAP93583.1 thioredoxin reductase
1.08	1.11	2.06	0.99	0.95	Tpx-5	>XP_395319.2 peroxiredoxin-6
0.97	0.03	1.03	0.06	1.01	Cyp4g11	>NP_001035323.1 cytochrome P450 4G11
0.97	0.30	1.32	0.35	1.02	LOC724870	>XP_016769012.1 NADPH–cytochrome P450 reductase
1.67	1.65	2.12	0.45	0.48	GstU1/No	>XP_001121730.2 glutathione S-transferase 1
1.51	1.35	2.52	1.01	1.17	LOC413043/No	>XP_396494.4 UDP-glucuronosyltransferase 1–3-like
2.07	1.00	2.69	0.62	1.69	No/No	>XP_016768325.1 S-formylglutathione hydrolase
1.88	1.42	2.78	0.89	1.36	LOC408368	>XP_391917.2 adenosylhomocysteinase
1.41	1.00	2.61	1.19	1.61	LOC551927	>XP_006567931.1 alcohol dehydrogenase [NADP(^+^)]-like
0.72	1.07	2.12	1.40	1.05	LOC408871	>XP_392401.3 sorbitol dehydrogenase-like
1.08	−0.62	2.48	1.41	3.10	LOC412231	>XP_623497.1 trans-1,2-dihydrobenzene-1,2-diol dehydrogenase-like
2.72	NaN	4.82	2.09	NaN	LOC411188	>XP_006569462.1 L-lactate dehydrogenase-like
−1.49	1.72	3.27	4.76	1.55	APD-3	>AER08515.1 apidermin 3-like protein

The log2 fold change differences between CON, DWV, VAR and DWV_VAR and gene names, including the indication of connections between markers in STRING, are shown. No means that the gene name was not found and/or connection in STRING was not indicated.

### Important protein groups

It is apparent that most of the various changes in the proteomes were indicative of upregulation by *DWV* signs and *Varroa* parasitism, and some of the changes were even amplified or, in contrast, reduced by the interaction between these two stressors. In a limited number of cases, we observed suppression of the protein abundance of some markers. The markers that specifically changed in DWV and those that changed similarly in DWV and DWV_VAR but not in VAR were considered highly specific to the effect of *DWV*. Analogously, the changes observed to be associated with VAR or both VAR and DWV_VAR but not DWV were considered specific to *Varroa*.

The influence of pathogens and parasites is connected to changes in the host immune system; thus, examining immune-related mechanisms is key for research on this subject. Therefore, we inspected honeybee immune genes. For this purpose, we first examined proteins listed in a review related to honeybee viral defense^21^; however, of the genes listed (see Table 1 in Brutscher *et al*.^21^), which we converted to protein equivalents (Table S1f), we identified 9 proteins in NCBI after a detailed investigation. The identification of only a small number of proteins is in part a consequence of the number of records removed from NCBI. Of these 9 proteins found in our study, 5 showed substantial changes (Table S1g). Although these markers were important for our story, it was necessary to investigate further. Therefore, we selected other important immune-related proteins from the list of identified proteins and found changes in a number of proteins involved in various immune pathways, i.e., JAK/STAT, MAPK, JNK, Toll, LLRs, NF-κB, RIG-I, and autacoid regulation. A recent study showed the upregulation of genes in the Toll, Imd, JAK-STAT, JNK and RNAi pathways in response to a virus^22^, and activation of the Toll pathway by *DWV* is believed to impair NF-κB signaling^8,9^. Another study showed that JAK-STAT, mTOR, MAPK and endocytosis genes were upregulated due to Israeli acute paralysis virus (IAPV)^23^. Furthermore, a metanalysis of transcriptomes showed that Toll and Imd pathway genes were differentially expressed after *Varroa*/virus infection^13^. Although we can find similarities to the markers expressed with *DWV* and *Varroa* among these studies, it is necessary to consider the honeybee stage at which analysis is performed as well as the experimental design^13^. Here, we analyzed the emerging bee, and therefore, the results should be unique to this stage.

When we investigated the changes in more detail, we were able to categorize the proteins into different functional groups (see Tables S1g–zh), which facilitated assessment. Because many proteins showed dramatic changes among variants, we worked with a threshold of at least a 0.8 log2 fold change when individually inspecting hundreds of key proteins. Final marker selection (markers with notes shown in Table S1zi) was used to generate (gene names provided in Table S1zj) a functional association network by STRING analysis, including some interconnected markers that changed to a lesser extent than a 0.8 log2 fold change. STRING analysis helped us generate conclusions about how key marker changes are connected by showing primary and linked markers. In brief, STRING analysis identified 284 of 336 selected proteins, for which the network (for stats, see Table S2) revealed key protein groups (Table S3) associated with the following KEGG pathways: metabolic pathways (Fig. S2); oxidative phosphorylation (Fig. S3), such as altered electron transfer chain (ETC) and ATP production; valine, leucine, and isoleucine degradation (Fig. S4), such as alternative ATP recruitment; fatty acid metabolism, degradation, and elongation (Fig. S5), such as the induction of fatty acids associated with virus replication; glutathione metabolism (Fig. S6), such as stress protection and virus replication; the Wingless (Wnt) and Hippo signaling pathways (Fig. S7), such as the regulation of organ growth; RNA transport (Fig. S8), such as virus replication; and others. However, some important markers, such as phospholipase A-2 (Pla2a), which is a key component in the arachidonic acid metabolism pathway (Fig. S9), could not be incorporated to interconnect their proteins into the current network version. Finally, the STRING analysis indicated the importance of the thioredoxin-like fold InterPro domain (Fig. S10). The selected markers that resulted from our analyses are listed in Table 1 and are further discussed.

### Excessive activation of the TGF-β pathway in the *Varroa*–honeybee–*DWV* interaction

In the latter investigation, we considered that *Varroa* saliva secretions impair host hemocyte-mediated wound-healing and plugging responses^24^. Among possible candidates, transforming growth factor beta 1 (TGF)-β1 or its analogs have been previously identified in tick saliva^25^. To ascertain whether there is a connection between the *Varroa*-induced protein changes observed in this study and TGF-β1, we compared our proteome-based changes with expression changes related to TGF-β1 treatment previously observed in a mouse mammary gland epithelial cell line^26^. Interestingly, we identified 51 unique homologous proteins in the VAR variant (compared via BLASTP) relative to those from a previous study^26^ in which 50 markers were analogously changed (for details, see Table S4). In DWV_VAR, 49 proteins changed in a similar manner, and the one protein that differed was Pla2a. However, in the DWV variant, only 43 markers were analogously changed. This result indicated the presence of TGF-β1 or its influencer in *Varroa* saliva. However, notably, viruses have also been shown to influence TGF-β1 by either promoting or suppressing the TGF-β signaling pathway^27,28^. The upregulated *DWV*-specific marker, which likely promotes TGF-β, is chloride intracellular channel exc-4 (exc-4/clic4)^29^. Furthermore, increases in AP-1-β1 and AP-1-mu-1, which were highest in DWV and DWV_VAR, can represent a marker associated with TGF-β activation of vascular endothelial growth factor expression^30^. Therefore, we suggest that *DWV* positively regulates TGF-β signaling and that *Varroa* and *DWV* can have a synergic effect on TGF-β pathway activation.

The TGF-β pathway is impacted through modulation of eicosanoid metabolism^31^. Interestingly, the key player in the eicosanoid pathway, Pla2a^32^, was specifically downregulated in DWV_VAR, implying the suppression of eicosanoid generation by arachidonic acid associated with infection and injury^32^ when *DWV* infection and *Varroa* parasitism of the host act together. Moreover, we suggest that the effect of Pla2 suppression can augment 15-hydroxyprostaglandin dehydrogenase [NAD^+^] (Hpgd), which is known to inactivate prostaglandins and/or lipoxins^33^. Hpgd upregulation was not specific to *Varroa* or *DWV*, but importantly, its upregulation in DWV_VAR amplified the Pla2-downregulation-induced suppression of inflammation specific to DWV_VAR. Furthermore, the effect of TGF-β1 on proliferation is enhanced by eicosanoid inhibition^31^. Thus, we suggest that, in addition to the activated TGF-β pathway, the suppression of the eicosanoid proinflammatory component of the innate immune response is a previously unknown key factor in the *Varroa* and *DWV* interaction.

TGF-β induction has been reported in some cases to stimulate^34^ or reduce^35^ synthesis of the basement membrane-specific heparan sulfate proteoglycan core protein, commonly known as perlecan. Perlecan, as a multifunctional component of the extracellular matrix, shows different effects on distinct cell types^36^. Because we observed *DWV*-specific upregulation and opposing *Varroa*-specific downregulation (almost qualitative) of perlecan, including in DWV_VAR, specific, context-dependent cell migration and proliferation regulation is suggested; for example, perlecan blockade inhibits proliferation of vascular endothelial cells, and exogenous perlecan promotes the proliferation of keratinocytes (see Nakamura *et al*.^36^). Perlecan is a known key factor in vascularization, sequestering growth factors such as fibroblast growth factor 2^37,38^, and its suppression by *Varroa* should thereby be connected to the wound-healing delay after injury^38^. In all treatments, the *Varroa*-induced perlecan effect can synergistically act with upregulated CD109 antigen, which inhibits the TGF-β1-induced-antiproliferative effect and wound closure^39^. Although CD109 attenuates TGF-β1 signaling in certain cells, it also promotes epidermal growth factor^40^.

### p38 modulation of p53 and excessive JAK/STAT signaling

At least one other important feature is prominently connected to the aberrant TGF-β signaling pathway. The aminoacyl tRNA synthase complex-interacting multifunctional protein 2 (Aimp2/p38) p53 proapoptotic factor^41^ is a mediator of TGF-β signaling known to suppress the proto-oncogene c-myc^42^. Upregulation of Aimp2/p38 in DWV and DWV_VAR indicates the specificity of the effect of this marker due to *DWV*, but its effect was more significant in the stressor interaction. p53 is known as a general repressor of RNA polymerase III (Pol III)^43^, but in our study, general upregulation of the DNA-directed RNA polymerase III subunit RPC1 (Polr3a) indicated the opposite as well as induction of the RIG-I pathway connected with the induction of type I interferon (IFN-I) and NF-κB^44^. Polr3a activity can be linked to upregulated JAK/STAT signaling as an antiviral response, so the virus needs to modulate the JAK/STAT pathway^45^. Therefore, we suggest that *DWV* modulation of JAK/STAT occurs through p38/p53. Furthermore, upregulated casein kinase II subunit alpha (CkIIalpha) likely plays a role in p53-mediated apoptosis, and its activity should be activated by p38 MAPK^46^. However, it is important to note that CkII is multifunctional and plays a crucial role in PI3K/AKT/mTOR, NF-κB, JAK/STAT and Wnt^47,48^. Additionally, several other kinases, i.e., protein kinase C (Pkc) and stress-activated protein kinase JNK (Sapk/bsk), are able to phosphorylate p53^46^ but are also of key importance in other pathways such as Wnt, which is discussed further. Notably, a number of other markers were indicative of p53 modulation, e.g., RANBP2-like and GRIP domain-containing protein 5/6 (RanBP2), SUMO-conjugating enzyme UBC9-B (Ubc9-b/lesswright), proliferating cell nuclear antigen (Pcna) and small ubiquitin-related modifier 3 (Smt3). While Aimp2/p38 upregulation was likely a response to the virus and seems to be disadvantageous for *DWV*, the upregulation of another marker, ubiquitin carboxyl-terminal hydrolase 5 (Usp5), may be favorable for the virus. We suggest that upregulated Usp5 is associated with p53 deactivation^49^.

p53 activity is connected to antiapoptotic Bcl-2^50^, which should mainly be downregulated in DWV and DWV_VAR as a result of increased levels of PRKC apoptosis WT1 regulator (Pawr)^51^. Furthermore, p53 is known to induce the expression of the proto-oncogene BCL-6, which in turn suppresses p53^52^, and the *Drosophila* homolog of BCL-6, known as *ken and barbie* (*ken*), modulates the JAK/STAT pathway^53,54^. In all investigated treatments, we observed excessive JAK/STAT signaling through high upregulation of the signal transducer and activator of transcription 5B (Stat5B, also known as stat92e). JAK/STAT signaling upregulation is the key feature of the antiviral response in honeybees^21–23^; however, the expression pattern of the Stat5B marker observed here suggests high JAK/STAT activation by *Varroa* as well and even attenuation of Stat5B in the stressor interaction. Furthermore, we mostly observed upregulated tetra-peptide repeat homeobox protein 1-like (tprx1) in DWV_VAR, which, according to a BLAST search, can also be annotated as BCL-6 corepressor-like protein 1-like (Bcl-6c), zinc finger protein 512B-like, or with a lower probability, cyclin-dependent kinase inhibitor 1 C (Cdkn1c/p57). We suggest the interesting idea that the marker tprx1/BCL-6c represses BCL-6^55^, thereby facilitating the activity of Stat5B, but the function of the putative BCL-6c must be investigated in future analyses. Importantly, BCL-6 exerts much stronger DNA binding than STAT and is able to repress transcription via STAT binding sites^56^. Opposing effects on target genes and inverse proportional expression by STAT5 and BLC-6 were observed^57^, further supporting our suggestion. Taken together, JAK/STAT was hyperactivated, and the p53-BCL-6 feedback loop was disrupted, so interestingly, p53 and JAK/STAT simultaneously had large impacts in DWV_VAR. Finally, we suggest that the p53 pathway impacts the JAK/STAT pathway via the STAT-masking mechanism^58^.

### Non-Smad pathways

Although TGF-β signaling induces the Smad2/3 pathway, it can also promote non-Smad pathways, such as MAPK/ERK (Ras-Raf-MEK-ERK), PI3K/AKT/mTOR and Rho-like GTPase^59,60^, and it is also linked to the markers described in the above section dedicated to p53. Importantly, non-Smad pathways modulate p53 activity; therefore, Smad and non-Smad pathways are integrated^61,62^. Furthermore, important MAPK/ERK markers, i.e., dual specificity mitogen-activated protein kinase kinase 3 and dual specificity mitogen-activated protein kinase kinase dSOR1^63^, seemed to be upregulated differently by *DWV* and *Varroa*. In this context, prosaposin, a relevant corresponding marker that was upregulated in DWV_VAR, activates MAPK pathways^64,65^ through G-protein signaling^64^; moreover, as part of the PI3K/AKT pathway, prosaposin induces Schwann cell survival^66^, which is an important feature of the response to viral and mite stress. The elevated G-protein-signaling in DWV_VAR nicely supports the upregulation of G-protein-signaling modulator 2. Furthermore, the activation of MAPK/ERK and PI3K/AKT/mTOR^67^ corresponded to the mostly specific or DWV_VAR-specific upregulation of Ras. The Ras proteins include ras-like protein 2, ras GTPase-activating protein-binding protein 2, and ras-related proteins Rab-7a, Ral-a, Rab-1A, Rab-14; nonupregulation of ras suppressor protein 1 is also important in this context. The Rab proteins are essential in vesicle intracellular transport, and upregulation of these markers supports increased endo/exocytosis^68^. We stress that Rabankyrin-5, a FYVE-finger effector of Rab5^69^, and Rab-7a were both upregulated in all treatments and should mostly be associated with increased endocytosis, an important part of the virus life cycle^70^. The next set of important markers represents the ras-related p21 GTPases Rac1 and Cdc42, for which rho GTPase-activating protein 44 (Gap44) can act as a Gap. The Gaps switch off signal transduction^71^, and therefore the downregulation of Gap44 in DWV_VAR led to the promotion of the Rho-like GTPase signaling pathway. Moreover, serine/threonine-protein kinase Genghis Khan, which functions as a downstream effector, Cdc42, and regulator of actin polymerization^72^ were upregulated in DWV_VAR. The upregulation of the regulator complex protein LAMTOR1 (p18/Lamtor1; late endosomal/lysosomal adaptor, MAPK and mTOR activator 1) indicated mTORC1 activation, mainly in the stressor interaction^73^. Importantly, mTORC1 activation is amino acid dependent^74^, and leucine is crucial for mTOR activation^75^, which we found to increase metabolism; this event is discussed further in relation to the manipulation of energy requirements. Lamtor1 upregulation is likely a response to ROS accumulation, which is connected to p53-dependent apoptosis^73^.

### Specific upregulation of RanBP3 and Src42A in the stressor interaction

Importantly, we observed DWV_VAR-specific upregulation of ran-binding protein 3 (RanBP3), which negatively regulates TGF-β signaling by interacting with Smad proteins^76^. However, RanBP3-mediated Smad2/3 nuclear export requires dephosphorylation by protein phosphatase 1 (Ppm1a)^76^, which should be obstructed by the upregulation of exc-4/clic4 in DWV_VAR^29^. Moreover, activation of the components of the non-Smad Ras and PI3K pathways signals RanBP3 phosphorylation and modulation of Ran-dependent nuclear transport^77^, and this event, including RanBP3 upregulation, can increase ribonucleoprotein nuclear transport, especially in the early and late phases of viral infection^78^. Next, we propose that a possible function of upregulated RanBP3 is to downregulate excessive JAK/STAT signaling in DWV_VAR through nuclear transport of the highly upregulated Stat5B^79^. RanBP3 is also known to function in the nuclear export of active β-catenin (*armadillo* in *Drosophila*), thereby leading to suppression of the Wnt pathway^80^. Finally, we indicate the disruption of the p53-BCL-6 feedback loop described above^52^, and we also suggest modulation of the p53-β-catenin feedback loop^81^, in which the RanBP3 marker may play an important role.

The next key marker that was specifically upregulated in DWV_VAR is tyrosine-protein kinase Src42A (Src42A), which is linked to many partners according to the STRING analysis (see Table S1zk); its activity can be associated with Ras, MAPK, JNK, Hippo including the PDZ domain, LLRs, cytokine receptors, serine/threonine-protein phosphatase 2 A, DE-cadherin, acetyl-CoA carboxylase, EF-hand calcium binding protein, and exc-4/clic4. We identified Src42A as a specific theoretical partner for Stat5B (see analysis in Table S1zk), and this connection suggested the modulation of JAK/STAT signaling in DWV_VAR due to the consequent upregulation of Stat5B and Src42A as a link between Src kinase activation and the function of Stat92E, which has also been indicated in *Drosophila*^82^. Importantly, Src42A has been described to regulate the JNK signaling pathway and epidermal closure in *Drosophila*^83,84^, and upregulation of Src42A should be specifically associated with the activation of inflammatory cell signaling pathways in wounds^85^. It is also likely that Src42A upregulation correlates with upregulation of proteins connected to the elimination of reactive oxygen species (ROS; see Table S1h), particularly H_2_O_2_, which is detected by Src42A^85^. Finally, Src42A has been described to act as a negative regulator of RTK/Ras/Raf/MAPK signaling^86^. Thus, it is likely that upregulated Src42A functions to inhibit wound-induced transcription in epidermal cells^87^.

### Wnt and Hippo signaling

Upregulation of Src42A correlates with Sapk/bsk activation^84^, and the STRING analysis (Fig. S7) indicated that Sapk/bsk is a shared protein in Wnt and Hippo signaling. DWV_VAR-specific upregulation of MOB kinase activator-like 1 (mats) functions as an activator of large tumor suppressor (Lats)/Warts (Wts) kinases in Hippo signaling, but it can be hypothesized that the additional effect of Src42A in DWV_VAR is to abolish the effect of mats^88^. Hippo and JNK signaling regulate wound-induced polyploidization (WIP), and JNK through AP-1 limits rather than stimulating Yki activation and polyploidization in the *Drosophila* epidermis^89^. Considering that the activated AP-1/JNK pathway can promote expression of proapoptotic genes^90^, the upregulation of AP-1-β1 and AP-1-mu-1 is an important result. In the case of Wnt, it is important to consider that the canonical Wnt is β-catenin-dependent and that the noncanonical planar cell polarity Wnt (Wnt/PCP) and Wnt/Ca^2+^ transduction cascades function independently of β-catenin^91^. Activation of Wnt/PCP is characterized by upregulation of Rac and JNK, Rho, and profilin, whereas the activation of Wnt/Ca^2+^ is characterized by the upregulation of Pkc, calcium/calmodulin-dependent protein kinase II (Camkii) and calcineurin^91^. Moreover, extrinsic Wnt is connected to the polarity component of atypical protein kinase C (aPkc)^92^, which was mainly upregulated in DWV_VAR. A gain in aPkc function has been shown to transform growth by disrupting Hippo/Yap (Yki) signaling^93^. This aberrant aPkc activity disrupts cell polarity, and its full activity requires partitioning of defective protein 6 (Par-6) containing the Cdc42- and Rac-interactive binding (CRIB)-PDZ domain^94^. Here, the PDZ-domain-containing uncharacterized protein LOC409904, which showed a similar expression pattern to that of Par-6, may also participate. Further, aPkc activity should be affected through its interaction with RanBP2/Nup358^95^, which was mainly upregulated in DWV_VAR.

The *DWV*-upregulated Camkii is also a Wnt signaling member. This multifunctional kinase is best known to affect learning and memory but is also linked to Wnt activation^96^ and can be connected to viral infection in the brain^97^, where *DWV* is localized^98^. Upregulation of calcyclin-binding protein in DWV_VAR enhanced the ubiquitin-mediated degradation of nonphosphorylated β-catenin^99^, and importantly, this event was connected to p53 activation observed here^100^. Rac1 is an important member of the Wnt signaling pathway that mediates β-catenin phosphorylation and stimulates the β-catenin-dependent transcription of Wnt target genes^101^; activation of Rac signal transduction was described above. Furthermore, coronin-1C upregulation supports Rac1 activation^102^ and is a marker of myosin II disassembly^103^. Additional markers include the upregulated Toll-like receptor (TLR) activator leucine-rich repeat flightless-interacting protein 2 (LRRfip2)^104^, which activates Wnt upstream of β-catenin^105^. Furthermore, slit homolog 1 protein-like has been described as a Wnt/β-catenin target^106^.

Considering the expression patterns of the markers, both canonical and noncanonical Wnt pathways were activated in the stressor interaction. Moreover, disruption of the crosstalk between Wnt and Hippo was indicated but may be cell specific. We note the recent finding that activated Hippo can paradoxically promote JNK-dependent cell migration^107^, but this event opposes macropinocytosis, which should result from the upregulation of aPkc and Src42A observed here^108^.

### Lethal(2)

The activation of Wnt/β-catenin signaling has been shown to be necessary for TGF-β-mediated fibrosis, but this activation is Smad independent and occurs through p38 MAPK^109^. Among the proteins showing the greatest changes in response to stressors was the upregulated protein lethal(2)essential for life (lethal(2)), which has an α-crystallin domain and is a member of the group of small heat shock proteins (SHSPs) that are induced by stress and are ubiquitous and analogous among vertebrates and invertebrates^110,111^. Recently, it has been suggested that lethal(2) has a function in antiviral defense^22^; however, we suggest that the strong increase in DWV and even its amplified expression in DWV_VAR are consequences of the stress-responsive MAPK cascade because the protein is activated by the p38 MAPK cascade and MAPKAP kinases. Additionally, and importantly, the expression pattern of lethal(2) was similar to that of Aimp2/p38. Furthermore, many studies have shown a connection between α-crystallin/SHSPs and ischemic injury^112–114^. The overexpression of lethal(2) in honeybees is likely associated with *DWV* symptoms and is further amplified by *Varroa*, which sucks the host and inhibits wound healing, thereby restricting the blood supply to tissues. Overall, one alternative non-Smad pathway targeting p38 results in the excessive production of lethal(2) as a corresponding marker.

### *Varroa* NF-κB downregulation overrides *DWV* activation

TGF-β1 has been reported to be an apoptosis activator that downregulates NF-κB^115^. However, NF-κB should be activated in response to infection by the virus, which hijacks NF-κB through modulation of various pathways^116^. Therefore, we inspected proteins connected to NF-κB regulation.

An important marker of the host inhibitory effect on *DWV* replication is upregulated hsp70-binding protein 1 (HspBP1), which inhibits viral gene expression mediated by NF-κB^117^. Thus, upregulation of HspBP1 in DWV and DWV_VAR may be considered a marker indicating virus suppression^117^ and this effect was more significant in the stressor interaction. Furthermore, suppression of NF-κB illustrated significant upregulation of the key marker NF-kappa-B inhibitor cactus (Cact2)^118^ in DWV_VAR. The association of Toll-mediated Hippo with downregulation of Cact2^119^ indicates the possible conflict between the effects of *Varroa* and *DWV* in the stressor interaction. We hypothesize that the abovementioned Src42A-abolishing effect of upregulated mats participates here as a Hippo component^88^. Furthermore, mitogen-activated protein kinase-binding protein 1 ( = JNK-binding protein; Mapkbp1 = Jnkbp1) should downregulate NF-κB^120^, but it can also activate JNK as a result of the TGF-β activation^121^ in VAR and DWV_VAR. In this connection, the inhibition of NF-κB in dendritic cells has been shown to induce strongly augmented JNK/AP-1 activity because of elevated levels of ROS^122^, illustrating the number of increased markers (see Table S1h). The elimination of dendritic cells is important in that it leads to immunologic indifference^122^. The expression patterns of AP-1s suggest that *DWV* drives immune indifference, but accounting for other relevant markers, the effect is greatest in the interaction with *Varroa*.

Although we first mentioned strong indicators of NF-κB suppression, other markers indicate the opposite; the aforementioned Bcl-2 suppression by Pawr should be connected to NF-κB activation^123^. The TLR-related upregulated proteins connected to NF-κB activation are LRRfip2^104^, dorsal protein isoform (dorsal-1A)^124^, and an evolutionarily conserved signaling intermediate in the Toll pathway (Ecsit)^125^. Additionally, Ecsit is required for bone morphogenetic proteins (Bmps), which are members of the TGF-β family^126^. Another important marker change indicating activation of NF-κB is Polr3a, which has been shown to induce IFN-I^44^. Furthermore, the function of Pol III is promoted by activated Ras signaling^127^. Upregulation of these markers was observed in all treatments, but similar to the markers of NF-κB suppression, it was most significant in DWV_VAR.

Aberrant NF-κB also has connections to the proteins responsible for chromatin remodeling, mainly by acetylation/deacetylation^128^. Among these, we encountered dynamic gene regulation following *DWV* infection and *Varroa* parasitization. According to the three established differentiating enhancer-binding transcription factor models^128,129^, we recognized two principally affected regulatory classes: (i) upregulated JNK/AP-1 contributed to the above discussed histone acetylases/deacetylases in local chromatin modification and remodeling, and (ii) chief inducible factors suppressed NF-κB and upregulated Stat5B. At this point, we linked the above discussed BCL-6c and Stat5B to NF-κB such that BCL-6 and NF-κB cistromes mediate the opposing regulation of the innate immune response^130^. Note that if BCL-6c targets BLC-6, its overexpression can represent an attempt to boost Smad signaling^131^. Moreover, the similarity to Cdkn1c/p57 suggests a link to TGF-β, i.e., the cyclin-dependent kinase inhibitor specifically induced by TGF-β should be required for cell cycle arrest^132^. Furthermore, it is imperative to note that in the *DWV* and *Varroa* interaction we observed upregulation of RanBP3, which should be able to transport Stat5B and downregulate excessive JAK/STAT signaling^79^. Finally, NF-κB is the common TLR mediator^130^. Therefore, according to our results, the TLR–NF-κB subnetwork in DWV_VAR is likely specifically influenced by the impaired BCL-6^130^.

Hyperactivated STAT in DWV_VAR should induce supercompetitor cells that kill the loser cells that function independently of Wnt, Yorkie and Myc^133^. However, highly elevated JAK/STAT in DWV_VAR may be the result of *Varroa* attempting to downregulate NF-κB through TGF-β1 or an analog^115^. Thus, it appears that *Varroa* and *DWV* are conflicting in their regulation of the NF-κB pathway via suppression or promotion, respectively, during their interaction. Although a negative effect of *DWV* on NF-κB was recently described using a few markers^8,9^, our high-throughput experimental approach using emerging bees is different from the approach utilized in that study.

We conclude that *Varroa*-induced NF-κB inhibition prevails on *DWV* infection-induced NF-κB activation in the DWV_VAR variant. The competition between NF-κB activation or suppression is connected to excessive JAK/STAT signaling, which can be diminished by the RanBP3 marker.

### Histone deacetylase Rpd3 as a conjunction marker

Based on the protein changes observed and the STRING analysis, we identified histone deacetylase Rpd3 (Rpd3) as a key marker. According to the STRING analysis, the function of HDAC1 ortholog Rpd3 is associated with upregulated Stat5B, Pcna, Ubc9-b/lesswright, Cact2, Polr3a, CkIIalpha, Rabankyrin-5, actin-related protein 1, enolase-phosphatase E1, downregulated Sumo3, and rather constant Arm/β-catenin. Considering the previous discussion, these members are associated with JAK/STAT, p53, NF-κB, RIG-I, PI3K/Akt/mTOR, Wnt, endocytosis, and amino acid metabolism. Similar to Rpd3/HDAC1, one of the above key markers, Src42A, is directly linked to Stat5B, Cact2, and Rabankyrin-5 according to the STRING network and thus has an additional impact on the corresponding pathways. Another important marker that is likely connected to Rpd3/HDAC1 is tprx1/Bcl-6c^55^. Although Rpd3 upregulation was not *Varroa*-specific, its effect was primarily highlighted by associated markers that were specifically or mostly changed as a result of the interaction between *DWV* and *Varroa*, in which the highest transcriptional activation by STAT was generated by chromatin remodeling via acetylation/deacetylation^134^. Similar quantitative changes to Rpd3 were observed for host cell factor 1, which has been described to activate viral gene transcription, scaffolding histone-modifying proteins and regulation of various cell cycle stages^135^. Thus, it is likely that hcf-1 influences transcription collectively with Rpd3. Overall, our results imply that histone posttranslational modifications drive the altered pathways. Relevant additional markers are, for instance, histone acetyltransferase type B catalytic subunit and protein arginine N-methyltransferase 1, which were mostly upregulated in DWV and DWV_VAR.

### Surprising upregulation of hexamerin 70c and 110 due to *Varroa* and the stressor interaction

Metamorphosis, which is characterized by total body reconstruction from a larva to an adult, is connected to extensive protein depletion until the first feeding after emergence^17^. The principal proteins used in metamorphosis are hexamerins^17^. Depletion of the storage proteins due to *Varroa* mite feeding on the host^17,136^ or increased demands for protein synthesis due to virus infection has been suggested; however, recent transcriptome studies did not report important changes in hexamerin expression in relation to virus infection and/or *Varroa* parasitism^4,22^. Thus, the strong upregulation of hexamerin 70c and 110 associated with *Varroa* parasitism and the upregulation associated with the combined effects of *DWV* and *Varroa* observed in this study are very interesting. The opposite situation was found for hexamerin 70b, which was downregulated by *DWV* and *Varroa* and to a lesser extent by their interaction. Moreover, downregulation was observed for hexamerin 110 in the DWV variant. These results suggest contradictory mechanisms of action by the investigated stressors affecting the abundance of these proteins, which serve as crucial protein sources during metamorphosis. The increased abundance of hexamerin c and hexamerin 110 may be an attempt of the host to compensate for the loss of the key proteins needed for successful transition to the adult stage. The finding that hexamerin 70a levels were almost constant in all investigated variants supports our previous suggestion that this protein is not a storage protein used during metamorphosis^17^. Overall, the highly contradictory changes in the storage of hexamerins 70c, 110, and 70b due to *DWV* infection and *Varroa* parasitization resulted in amplification of the effects of *Varroa* parasitization, with this interaction likely leading to a fatal retarding effect on development.

### MRJPs, vitellogenin, and JH-related proteins

Additional nutrition-related proteins are the major royal jelly proteins (MRJPs)^137^ and the reproductive protein vitellogenin^138,139^. However, and importantly, these storage proteins are also linked to immunity^137,138,140^. The results of this study showed upregulation of MRJPs in DWV, VAR, and DWV_VAR and downregulation of the vitellogenin precursor, mainly in DWV and DWV_VAR. The higher content of MRJPs, especially MRJP1 and MRJP3, is likely related to immune defense^140^. Interestingly, MRJP1 and MRPJ3 are also present in the honeybee brain^141^.

Importantly, low vitellogenin levels are connected to higher levels of juvenile hormones (JHs)^139^. The strong decrease in vitellogenin levels in DWV and DWV_VAR correlated to increased levels of farnesol dehydrogenase-like, which are connected to JH synthesis^142^, but upregulation of JH epoxide hydrolase 1, which inactivates JHs^143^, seems to be a counteracting mechanism and an attempt to decrease JH levels. Importantly, a decrease in vitellogenin has been found to be associated with apoptosis of hemocytes; this mechanism to decrease immunity has been described for worker bees, which dramatically downregulate their defense machinery when they switch from being hive workers to foragers^144^. Thus, the results indicate that vitellogenin suppression is driven by *DWV* to prevent viral destruction by hemolymph-based immune protection. This mechanism is even amplified by the interaction of *DWV* with *Varroa*, but it does not seem to be initiated by the parasite alone.

### Manipulating energy requirements

The increase in energy requirements resulting from viral infection is met by mitochondria. The upregulation of various proteins of the ETC (Table S1i), including the electron transfer flavoprotein (ETF), was consistent with the observation that viruses direct mitochondria to produce more ATP to supply energy^145^. Based on that connection, we further identified the strong upregulation of an isovaleryl-CoA dehydrogenase, mitochondrial (IVD), which is a mitochondrial matrix enzyme that catalyzes the third step in leucine catabolism, providing electrons to ETF^146^. Thus, high IVD upregulation is likely a mechanism connected to the increased need for ATP during nutrient starvation^147^. Moreover, IVD could hypothetically regulate mTORC1 activation by affecting leucine metabolism^75^. Finally, IVD upregulation may also be important for eliminating toxic isovaleryl-CoA^148^. Overall, we suggest that the elevated expression of IVD in response to *DWV* and *Varroa* plays a role in cell-mediated immunity against pathogens and consequently is an alternative generator of ATP in a starved, metamorphosing honeybee. One of the markers that should affect mitochondrial function is the abovementioned Ecsit^149^; we suggest a possible link between this TLR/BMP pathway-related marker and upregulated oxidative phosphorylation, the inflammatory response, and developmental changes^149^.

### ROS elimination and detoxification – distracted metabolism responses

Pathogen infection is connected to increased production of ROS, which kills pathogens directly or through signaling pathways that activate the immune response, including the NF-κB pathway^150^. In our study, various proteins associated with ROS (Table S1h) reduction were upregulated, mainly thioredoxin-related proteins and peroxiredoxin-6. Therefore, ROS elimination due to *DWV*, *Varroa*, and their interaction seems to be primarily connected to these pathways.

Among the classes of proteins involved in detoxification (Table S1j), similar increases in the coexpressed cytochrome P450 4G11 (Cyp4g11) and NADPH-cytochrome P450 reductase in DWV and DWV_VAR are likely connected to the altered expression of cuticular hydrocarbons^151^. This observation is consistent with the effects on many proteins related to the cuticle (Table S1k). Next, among the glutathione S-transferases and UDP-glucuronosyltransferases that showed changes in expression, the greatest changes were observed for glutathione S-transferase 1 and UDP-glucuronosyltransferase 1–3-like, which were both increased similarly in DWV and VAR and were even amplified in DWV_VAR. This finding suggests that these proteins play important roles in the detoxification of endogenous compounds related to virus infection and *Varroa* parasitism.

An interesting finding related to detoxification was the upregulation of S-formylglutathione hydrolase (Fgh), which is an esterase D that is involved in the detoxification of formaldehyde^152^. Similar upregulation was observed for alcohol dehydrogenase [NADP(^+^)]-like (Adh). Since adenosylhomocysteinase controls methylation through regulation of intracellular adenosylhomocysteine (SAH)^153^, an increase in its levels should correlate with methanol and, consequently, formaldehyde production^154^. An increase in Adh may correlate with NADPH-dependent aldehyde elimination of alcohols^155^. According to the STRING analysis, Adhn activity is associated with other highly changed carbohydrate metabolizing enzymes, i.e., sorbitol dehydrogenase-like (Sdh) and trans-1,2-dihydrobenzene-1,2-diol dehydrogenase (Dhdh), and through sorbitol dehydrogenase, there is a further connection with L-lactate dehydrogenase (L-ldh) and Hpgd. The activated formaldehyde elimination pathway suggests an adaptation to formaldehyde resistance^156^; this adaptation may be attributed to aldehyde accumulation due to injury^157^. Upregulation of Sdh indicates activation of the polyol pathway, which is connected to insufficient insulin, ROS increases, and activation of MAPK and NF-κB^158^. Additionally, Sdh is an important marker affecting injury^159^. We emphasize that the activated polyol pathway, which has a reduced ability to regenerate glutathione due to NADPH consumption, leads to a decrease in the defense against oxidative stress^160^. Dhdh is an important detoxification enzyme that can regulate ROS generation and the apoptotic pathway response^161^, which, based on its expression, was specific to *DWV*. The elevated qualitative/quantitative levels of L-ldh in DWV_VAR were consistent with the finding that this enzyme is a known marker of ischemic damage^162^. Thus, these enzyme markers merit further investigation because they illustrate the distracted metabolism connected to immune system disruption and injury.

### Apidermin 3

Important changes representing possible conflicts between the effects of *DWV* and *Varroa* are indicated by apidermin 3-like protein, which was downregulated in DWV but upregulated in VAR and even in DWV_VAR. Unfortunately, little is known about apidermin 3, a cuticular protein that is expressed in the exoskeletal epidermis and is uniquely associated with nonpigmented cuticles such as the eye cover and external cuticle of white pupae^163^. Due to the limited information available, apidermin 3 stands alone in our story.

### *DWV* in the investigated samples – (un)certainty of detection

On average, the results of the label-free nLC-MS/MS proteomics analysis indicated the highest virus load in the DWV variant (see protein hits in the Table S1zm), while in the remaining sample types (including control) it was similarly low. We stress that it is necessary to carefully consider the proteomic detection of pathogen proteins in the host to prevent the pitfalls in data evaluation^5,164,165^. Therefore, to verify the accuracy of *DWV* identification in the bee samples, we extracted the peptides related to the *DWV* polypeptide identification. Overall, we recognized 36 different peptides identifying the DWV-polypeptide (see Table S1zn). Analysis of these peptide sequences and alignment to the divided polypeptide^5,166^ revealed that the peptides identified were both structural and nonstructural of the *DWV* polypeptide (see Table S1zn), indicating the *DWV* replication state^5^. The results show that the highest diversity of peptides identifying *DWV* was in the DWV variant; however, in the remaining sample types CON, VAR and DWV_VAR, quantitative identification of *DWV* was not reliable. Thus, in the future, it will be necessary to perform detailed quantitative analysis including qPCR on the presence of *DWV* in the emerging bees. Furthermore, it will be necessary to consider the influence of *Varroa* on *DWV* strain diversity^2,4,167^, which was not investigated in this study.

### Summarization

In summary, using label-free proteomics, we demonstrated that the proteome changes in the interaction of *Varroa* and *DWV* were directed by *Varroa* (Fig. 2). It appears that *DWV* and *Varroa* influence biochemical pathways by analogous and opposing transitions, which is somewhat consistent with the concept ascertained in the tick–host–pathogen interaction^19^. We stress one important objection to the comparison to ticks in our study, which is that multiple *Varroa* parasitize a single infirm bee in a capped cell. Therefore, the bee is pricked many times by *Varroa*, and the changes caused by this stressor become more obvious. One can imagine that similar effects can be achieved in humans parasitized continuously by hundreds of ticks. Moreover, the specific impact of *Varroa* is that parasitism occurs during metamorphosis and the bee cannot eat, all underlined by that the mite is tightly associated with *DWV* transmission. The analogy to ticks is also supported by our finding that *Varroa* saliva contains tissue growth influencers^25^. We proved the impact of *Varroa* on TGF-β signaling, which impairs host immunity.

**Figure 4:**
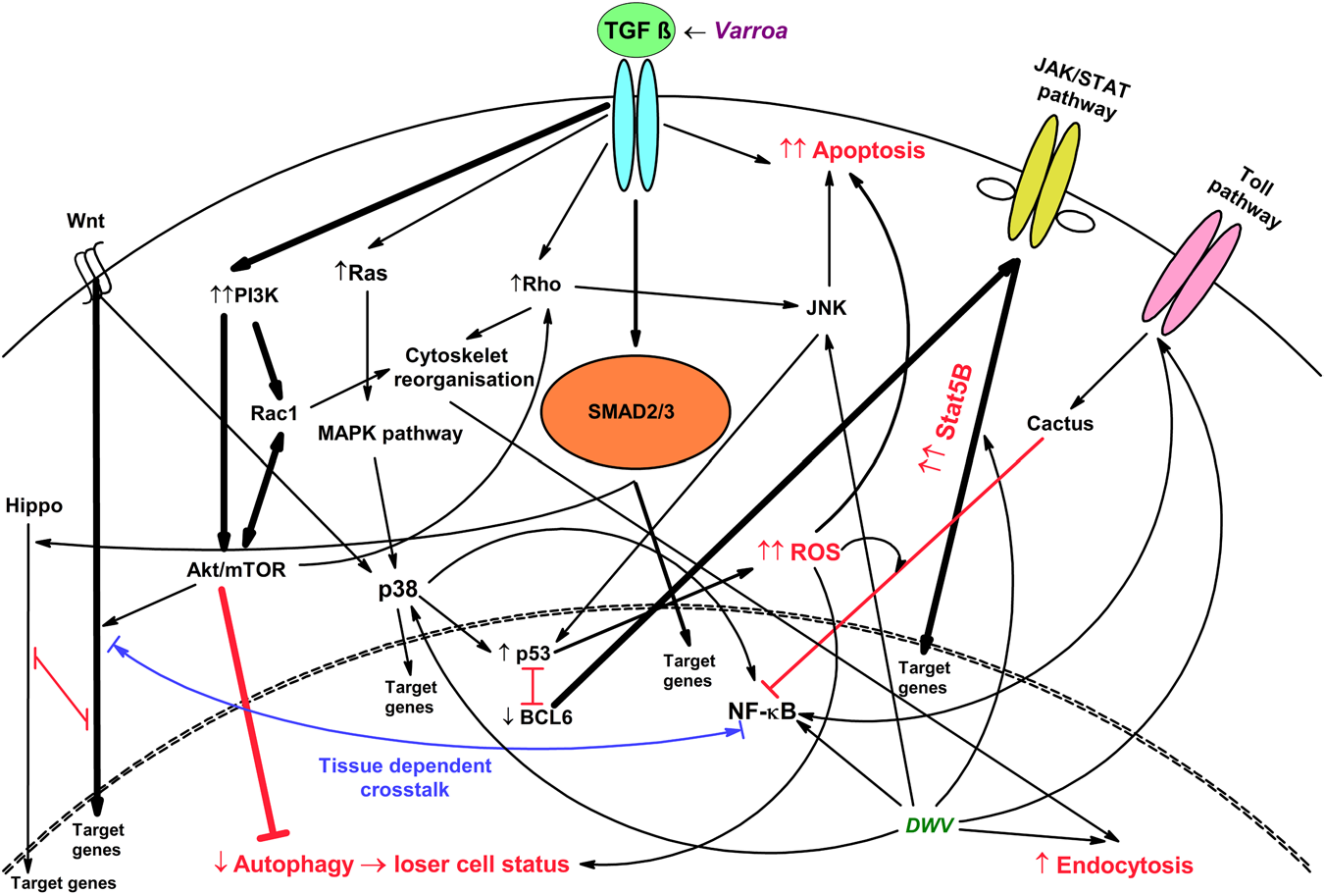
Simplified schematic representation of the affected pathways in an emerging honeybee worker that impact *Varroa* and *DWV* symptoms via their interactions. Multiple markers indicated that *Varroa* mite salivary compounds promote TGF-β signaling, which strengthens Smad2/3 pathway signaling, but alternative signaling pathways are propagated. Connection of the activated Wnt and Hippo pathways, which undergo crosstalk disruption, leads to aberrant tissue growth, which is the likely cause of the increased probability of wing deformity. Considering that *Varroa* overrides *DWV*-induced proteome changes, as shown in Fig. 2, this suggests that the mite suppresses mechanisms that normally facilitate *DWV* infection. Thus, there are conflicts in JAK/STAT and NF-κB signaling. We indicate the collective effect of *Varroa* and *DWV* on JNK, the domination of p53-induced apoptosis and that JAK/STAT is hyperactivated and the p53-BCL-6 feedback loop is disrupted. The cooperating effect is the facilitation of viral endocytosis by the mite. The activated Akt/mTOR (through upregulated p18/Lamtor1) pathway inhibits autophagy and recycling of cells damaged by ROS, which are activated by p53. The high number of damaged cells decreases the efficacy of ATP acquisition, resulting in loser cell status.

Based on proteomic changes, which were thoroughly inspected individually, we provide a simplified schematic illustration of the impacted biochemical pathways in the *Varroa*–honeybee–*DWV* interaction (Fig. 4). We indicate that *Varroa* and *DWV* compete to manipulate the immune system. Importantly, we note that *Varroa* and *DWV* have conflicting effects on activation and suppression of NF-κB, respectively. During their interaction, eicosanoid synthesis is suppressed, which augments the TGF-β pathway. We indicate that JAK/STAT is hyperactivated and that the p53-BCL-6 feedback loop is disrupted, so p53-induced apoptosis and JAK/STAT act simultaneously. Excessive JAK/STAT signaling likely occurs through the collective effect of stressors on JNK, i.e., TGF-β induces JNK and JNK activation due the host response to *DWV*. We hypothesize p53-dependent masking of STAT^58^ and suggest that the prevailing effect of RanBP3 in the interaction is not to diminish the Smad-dependent TGF-β pathway but to participate in viral replication and diminish excessive JAK/STAT and/or Wnt signaling.

Although *Varroa* and *DWV* cooperate in some respects, they have opposing effects on NF-κB and JAK/STAT. In the stressor interaction, the markers indicate facilitated entry of viruses into cells, which, compared to simple transmission, is a case of cooperation.

This study provides a set of important markers that should be further investigated in particular situations. Because our study was conducted at the time of honeybee emergence, the time at which the mite effect wins over *DWV* remains undetermined.

## Materials and Methods

### Study design

For the study presented in this paper, it was pivotal to show whether mites and/or signs of *DWV* occurred in the capped cells. The sample design (see Fig. 1) was selected to determine the interaction between the *Varroa* mite and *DWV*. Thus, the collected samples comprised the following four bee variants collected at the time when they were just emerging, i.e., when they started opening the capped cells: (i) control bees that were not parasitized by *Varroa* in the capped cells and had no recognizable *DWV* impact (marked as “CON”); (ii) bees parasitized by the *Varroa* mite in the capped cells but with no visible *DWV* impact (marked as “VAR”); (iii) bees parasitized by the *Varroa* mite in the capped cells and showing visible signs of DWV impact (marked as “DWV_VAR”); and (iv) bees impacted by *DWV* but not parasitized by *Varroa* in the capped cells (marked as “DWV”). Note that in the study design, sample (iv) served as the second control, along with the healthy bees (i). Furthermore, note that the DWV variant was difficult to find relative to DWV_VAR, suggesting that the mite could parasitize bee larvae and that nurse bees could remove it before capping; therefore, the mite was not present in the capped cells.

### Bee samples

The honeybees originated from the Bee Research Institute at Dol, Czechia. The samples, including controls, were collected from colonies infested by *Varroa* and with manifestations of *DWV* clinical signs. All samples were from naturally *Varroa* heavily infested colonies and had the same bee genetics, and no manipulative experiments were performed in this study. The samples were collected from 3 colonies and randomly selected for analysis. The honeybee workers were collected just as they started opening the capped cells. Four people attended the collection of samples from the comb: one person collected the bees with sterile tweezers and placed them into 0.5 mL Eppendorf^®^ LoBind tubes (Cat No. Z666491, Sigma-Aldrich, St. Louis, MO, USA), two others verified the number of mites present in the cells, and a fourth person recorded the data. The honeybee brood comb was taken from the *Varroa*-infested colony, and the collection time was limited to ca. 20 min to minimize the impact of the colony environment absence; then, the comb was placed back in the hive. The emerging bees were collected, and the mites, if present in the cells, were counted to obtain sample No./number of mites: 1/0, 2/0, 3/9, 4/7, 5/7, 6/12, 7/5, 8/4, 9/4, 10/7, 11/0, 12/0, 13/0, 14/0, 15/0, 16/0, 17/0, 18/0, and 19/5. Upon subsequent checking (Multiple Comparison p-value adjusted by Hochberg’s method^20^, all p-values > 0.5), there were no significant differences among samples with different mite numbers^168^.The collected samples were transported to the laboratory in a rack on dry ice and further stored in a deep freezer at −80 °C until use.

### Proteomic analysis

The emerged individual bees were homogenized using a 2 mL Potter-Elvehjem tissue grinder (Kartell Labware division, Noviglio, Italy) and drilling machine (PSB 650RE, Bosch, Stuttgart, Germany). In brief, each bee was initially homogenized in 0.5 mL of 50 mM Tris-HCl, pH = 7.4 with 1% Triton X-100 and an EDTA-free protease inhibitor cocktail tablet (Cat No. 05 056 489 001, Roche, Indianapolis, IN, USA) dissolved in 30 mL of buffer. The sample was initially homogenized for 1 min, left on ice for 10 min, homogenized again for 1 min and then a final time after incubation for 15 min on ice in 2 mL of nonpure water (ddH_2_O; Thermo Fisher Scientific, Waltham, MA, USA). The samples were centrifuged at 20,000 × g for 20 min at 4 °C in an MR 23i centrifuge (Jouan Industries, France). The samples were then processed analogously to FASP, which is available online^169^.

All samples digested with trypsin were analyzed by nLC-MS/MS in two analytical replicates. All 19 biological samples were analyzed in one analytical series; thus, 38 nLC-MS/MS runs were performed consecutively. First, one test nLC-MS/MS analysis marked as 1_1 was performed; thus, the analyses of sample 1 included in the dataset are marked 1_2 and 1_3, and the rest of the analyses are marked X_1 and X_2 (where X is sample No. 2–19). Note that we originally selected 20 biological samples, with 5 biological replicates for each treatment, but the last two analyses in the series of 40 analyses lacked spectra and therefore were not evaluated. Nano reversed-phase columns (EASY-Spray column, 50 cm × 75 µm ID, PepMap C18, 2 µm particles, 100 Å pore size**)** were used. Mobile phase buffer A was composed of water, 2% acetonitrile and 0.1% formic acid. Mobile phase B was composed of 80% acetonitrile and 0.1% formic acid. The samples were loaded onto a trap column (Acclaim PepMap300, C18, 5 µm, 300 Å Wide Pore, 300 µm × 5 mm) for 4 min at 15 μL/min, and the loading buffer was composed of water, 2% acetonitrile and 0.1% trifluoroacetic acid, with analysis in a Thermo Orbitrap Fusion Tribrid mass spectrometer (q-OT-IT) instrument performed as previously described^170,171^.

### Proteomic data evaluation

The data were analyzed and quantified with label-free algorithms using MaxQuant software (version 1.5.3.8)^172^. The FDR was set to 1% for both proteins and peptides, and we specified a minimum length of seven amino acids. The Andromeda search engine^173^ was used for the MS/MS spectra search against the NCBInr database downloaded on January 31, 2017. The actual database consisted of 150,263 records and contained not only the *Apis mellifera* genome but also pathogens (importantly viruses) and symbionts. The enzyme specificity was set as C-terminal to Arg and Lys, also allowing for cleavage at proline bonds and a maximum of two missed cleavages. MethylThio was selected as a fixed modification, and N-terminal protein acetylation and methionine oxidation were selected as variable modifications.

### Statistical data evaluation

The proteomic data evaluated via MaxQuant^172^ were further processed in Perseus (version 1.5.2.4)^174^. Contaminants, reverse, and only identified by site hits were removed, and the matrix was reduced to at least two positives in at least one group. The protein abundance data of intensities were logarithmically transformed as suggested by Anderson *et al*.^175^. We preferred log2 transformation, which permits better visualization of less-abundant species. The data were further analyzed using RDA and multiple comparison statistics using the “vegan” R package^168,176^. We evaluated whether it was beneficial to present our data including the technical replicates of the nLC-MS/MS analyses, because it allows us to show all variances influencing the results. Furthermore, this means of data presentation does not influence the statistical results from a mathematical perspective^168^. Distribution of the protein abundance to create the heatmap was computed from the abovementioned RDA analysis using the “predict” function in the “vegan” package^176^. The corresponding heatmap depicting the protein abundance and clustering in dendrograms was produced using the “gplots” R package^177^. In addition, we created a heatmap in the Perseus^174^ environment. The missing values were replaced based on a normal distribution (width: 0.3; down shift: 1.7; total matrix) and the data were normalized through the Z-score. Hierarchical clustering was performed using the average Euclidian distance (Preprocess with k-means; 300 clusters) for both row and column trees. The resulting heatmap was adjusted to grayscale. While the first heatmap is provided in the supplementary material, the latter is shown in the main manuscript; however, both heatmaps showed the same hierarchical clustering in columns.

### Individual data evaluation

We individually inspected the proteins that showed the greatest changes. We worked with the threshold, which was characterized by at least a 0.8 log2 fold change, but we enriched the final list of markers with some interconnected markers that changed to a reduced extent or did not change. Note that some markers are important just because they do not change, while the other do. We further grouped the proteins based on their function. The selected proteins were searched using UniProt and NCBI, and the description, selected biological and/or molecular function or protein region or each protein was linked to the description to facilitate characterizations.

### Data evaluation using STRING

To ascertain the functional association network, we identified genes for the selected proteins and entered them into STRING 10.5^178^. From the STRING network, we further selected the key KEGG pathways. It is important to note that not all proteins could be connected in STRING, but the analysis facilitated data evaluation.

### Search for proteins that are influenced by TGF-β

*Varroa* parasitization is likely to affect host wound healing. However, whether this phenomenon occurs during the *Varroa*-honeybee interaction remains unknown. Therefore, we searched previously described tick salivary compounds, which have been reviewed^25^. Among the possible candidates, we selected tissue growth factor influencers^179^. This selection was facilitated by the observation that tissue growth factor influencers have been reported to interact with our key selected markers. Among the factors inhibiting healing, we identified TGF-β^25^. We further searched the literature related to protein changes caused by TGF-β and found a relevant publication by Xie *et al*.^26^, which we used to compare markers and changes. Each sequence of the protein markers presented in Tables 1 and 2 in Xie *et al*.^26^ was processed via BLASTP^180^, and the list of proteins obtained using this process was searched for *A*. *mellifera* homologs. When the identified honeybee homologs displayed a homology higher than 50% and the same function, we determined whether the changes in the honeybee proteome corresponded to those reported by Xie *et al*.^26^.

## Supplementary information

Tables S1a–Szn

Supplementary Material

## Data Availability

The accession number for the Raw LC-MS/MS runs and searching database reported in this paper is MassIVE MSV000083681 10.25345/C57H0V or PXD013491.
