# Supplementary material for: Varroa destructor parasitism has a greater effect on proteome changes than the deformed wing virus and activates TGF-β signaling pathways
Source: Sci Rep. 2019 Jun 28;9:9400. doi: 10.1038/s41598-019-45764-1 (PMC6599063; doi:10.1038/s41598-019-45764-1)
Supplement: Supplementary file 2 — Supplementary Material [file 41598_2019_45764_MOESM2_ESM.pdf]

Supplementary Materials

Journal: **Scientific Reports**

Varroa destructor parasitism has a greater effect on proteome changes than the deformed wing virus and activates TGF-β signaling pathways

T. Erban,* B. Sopko, K. Kadlikova, P. Talacko & K. Harant

*Corresponding author. E-mail: arachnid@centrum.cz
ORCID iD: https://orcid.org/0000-0003-1730-779X

This PDF file includes:

Supplementary Results
Figs. S1 to S10
Tables S2 to S4
Reference List to this Content.

Other Supplementary Material for this manuscript includes the following:

The **Tables S1a–zn** are available in one condensed supplementary Excel file.

Supplementary Results

Results of the RDA analysis

Fig. S1 shows the statistical importance of the three RDA factors explaining the data variance. The scores of the RDA1, RDA2 and RDA3 factors obtained from the RDA analysis explain 65.83, 21.83 and 12.34%, respectively, of the variability in protein abundance of the measured proteomes. The RDA biplot (Fig. 2 in the main text) demonstrates the proteome changes in response to *Varroa*, *DWV* and their interaction. The detailed description is presented in the main text of the manuscript.

Figure S1. Visualisation of the scores of RDA factors obtained.

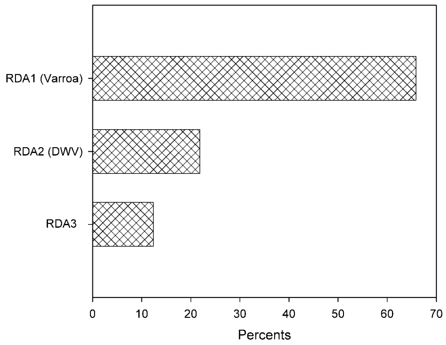

Cluster analysis of RDA factors

The cluster analysis of the RDA factors is helpful in explaining the observed changes. However, it is necessary to note that further manual data evaluation is required to seriously analyze the observed protein changes. The Table S1zl shows the clustered output of the RDA analysis using the "predict" function in the "Vegan" package[176].

STRING analysis

Overall, 284 of 336 proteins listed in the Table S1zj were analyzed by STRING[178]. Table S2 shows the STRING statistics, Table S3 shows the indicated KEGG pathways and Figs. S2–S9 illustrate the relevant proteins in the STRING network. Note that the STRING analysis facilitates the selection of the key protein markers and additional proteins can be identified in the literature. The STRING analysis indicated 11 members of thioredoxin-like fold INTERPRO domain (Fig. S10) in the gene set.

Table S2. Network stats of STRING analysis.

Network stats	
number of nodes:	294
number of edges:	1128
average node degree:	7.67
avg. local clustering coefficient:	0.447
expected number of edges:	802
PPI enrichment p-value:	< 1.0e-16

Table S3. KEGG pathways of the STRING analysis.

Pathway ID	Pathway description	Count in gene set	False discovery rate	Suppl. figure
00190	Oxidative phosphorylation	19	3.42e-11	Fig. S3
01100	Metabolic pathways	51	3.42e-11	Fig. S2
00280	Valine, leucine and isoleucine degradation	7	0.000345	Fig. S4
01212	Fatty acid metabolism	7	0.000425	Fig. S5
00071	Fatty acid degradation	6	0.000726	Fig. S5
00480	Glutathione metabolism	6	0.000788	Fig. S6
00310	Lysine degradation	6	0.000868	
04310	Wnt signaling pathway	8	0.000948	Fig. S7
00640	Propanoate metabolism	5	0.00103	
00062	Fatty acid elongation	4	0.00112	Fig. S5
00380	Tryptophan metabolism	5	0.00112	
03013	RNA transport	11	0.00112	Fig. S8
04391	Hippo signaling pathway - fly	7	0.0013	Fig. S7
00270	Cysteine and methionine metabolism	5	0.00445	
01120	Microbial metabolism in diverse environments	9	0.00445	
00980	Metabolism of xenobiotics by cytochrome P450	3	0.00845	
01200	Carbon metabolism	7	0.0195	
00650	Butanoate metabolism	3	0.0208	
00590	Arachidonic acid metabolism	3	0.0257	Fig. S9
00020	Citrate cycle (TCA cycle)	4	0.0443	

Figure S2. STRING pathway proteins: metabolic pathways.

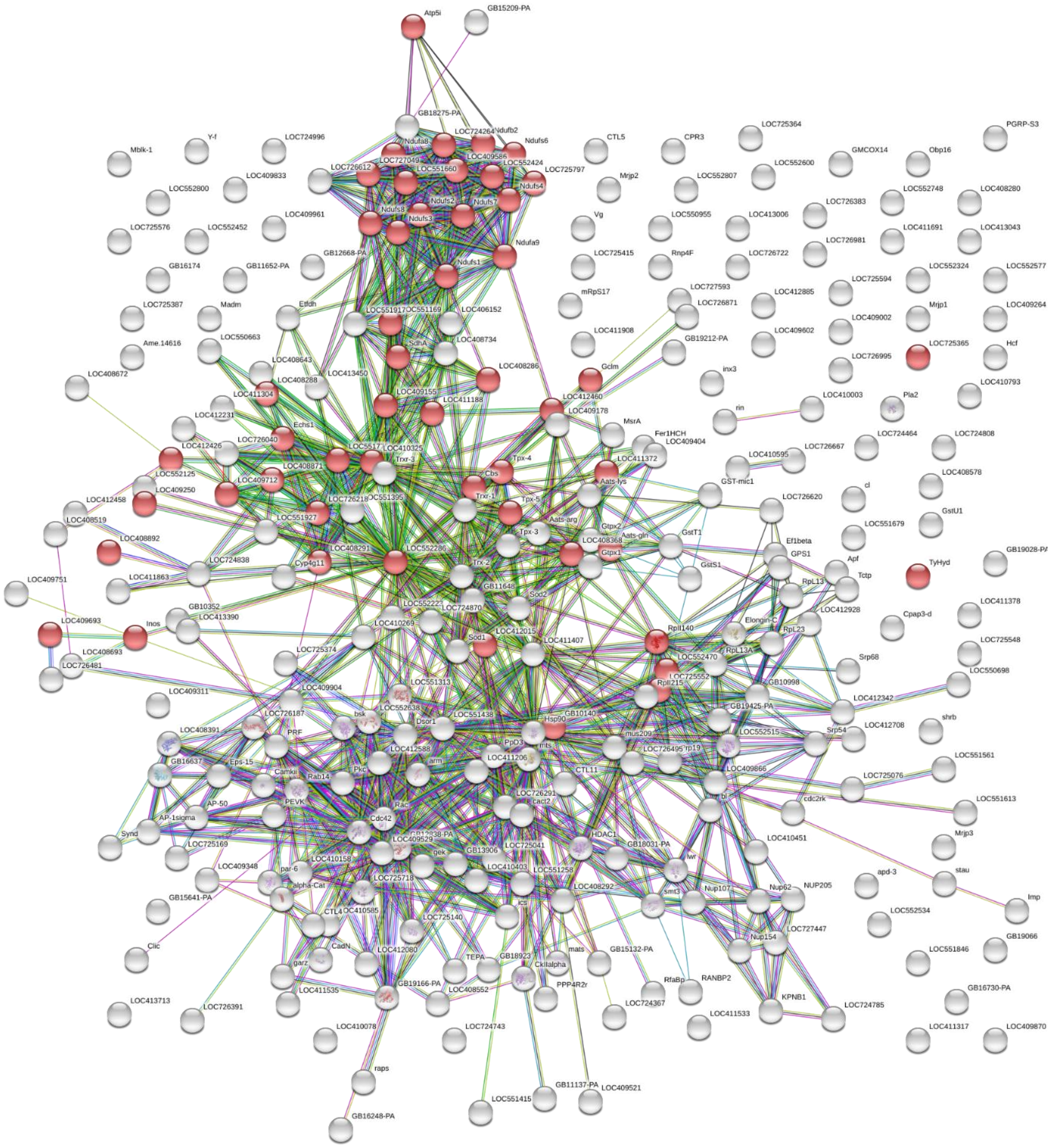

Figure S3. STRING pathway proteins: oxidative phosphorylation.

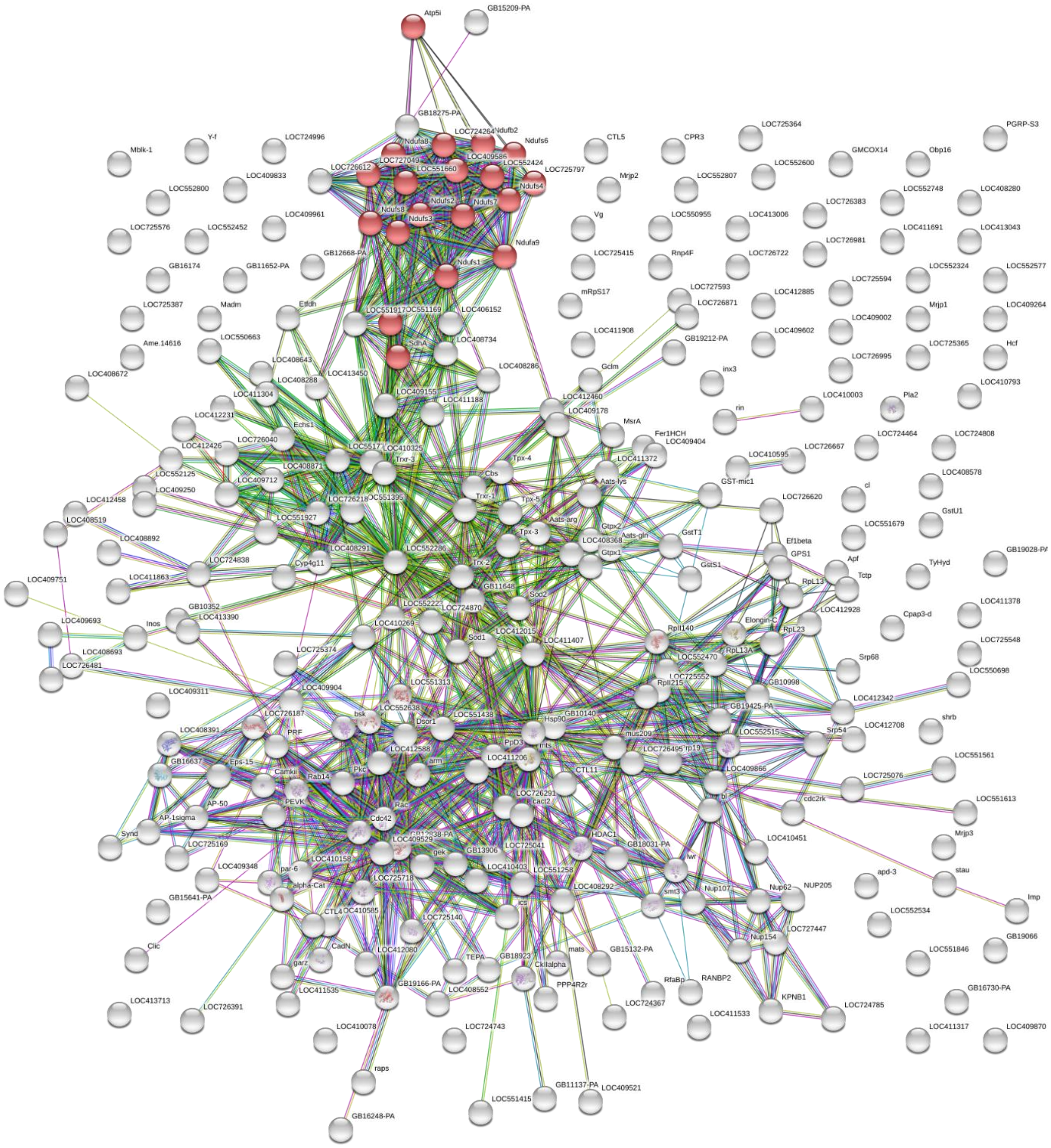

Figure S4. STRING pathway proteins: valine, leucine and isoleucine degradation.

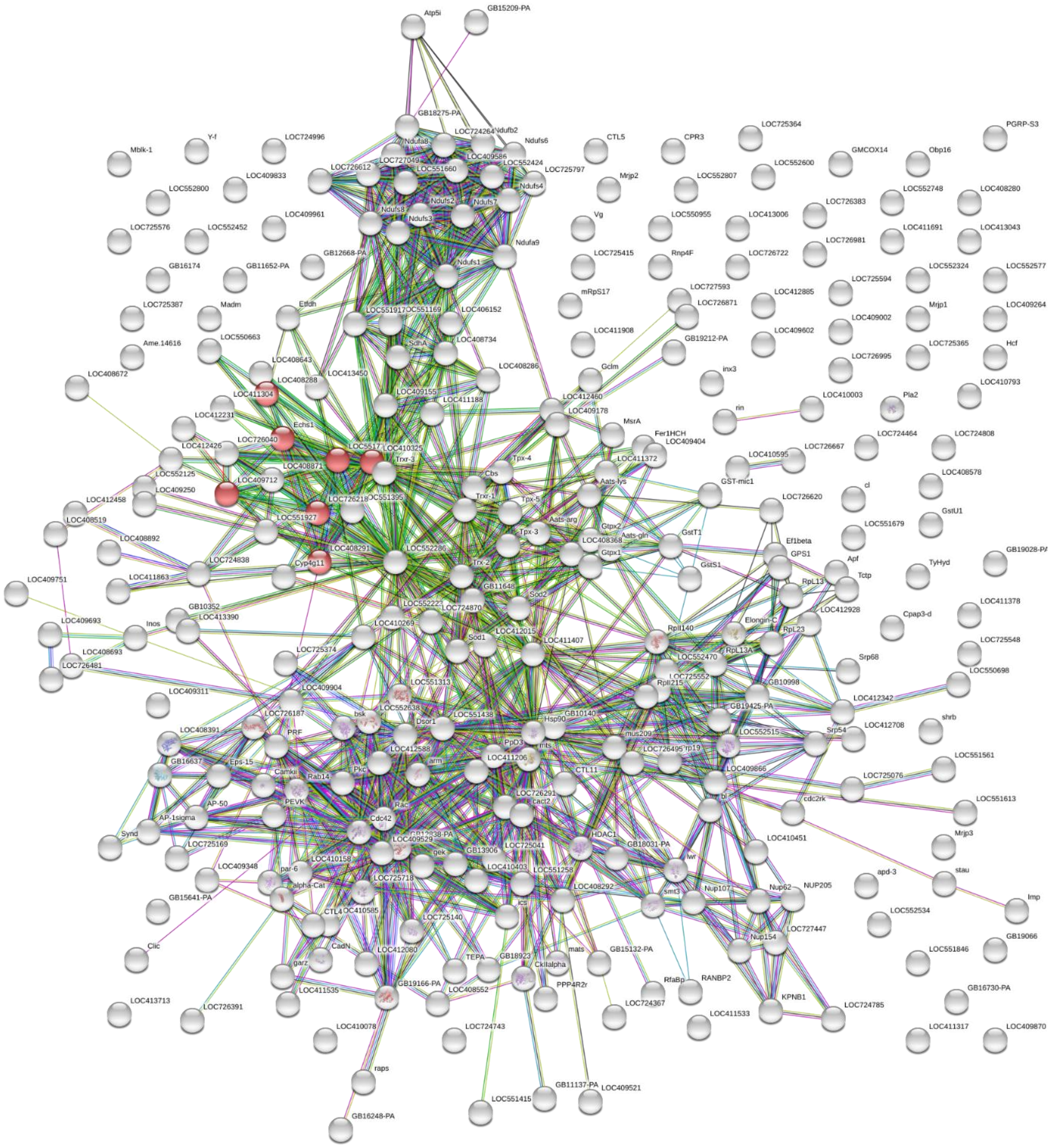

Figure S5. STRING pathway proteins: fatty acid metabolism (red), degradation (blue), and elongation (green).

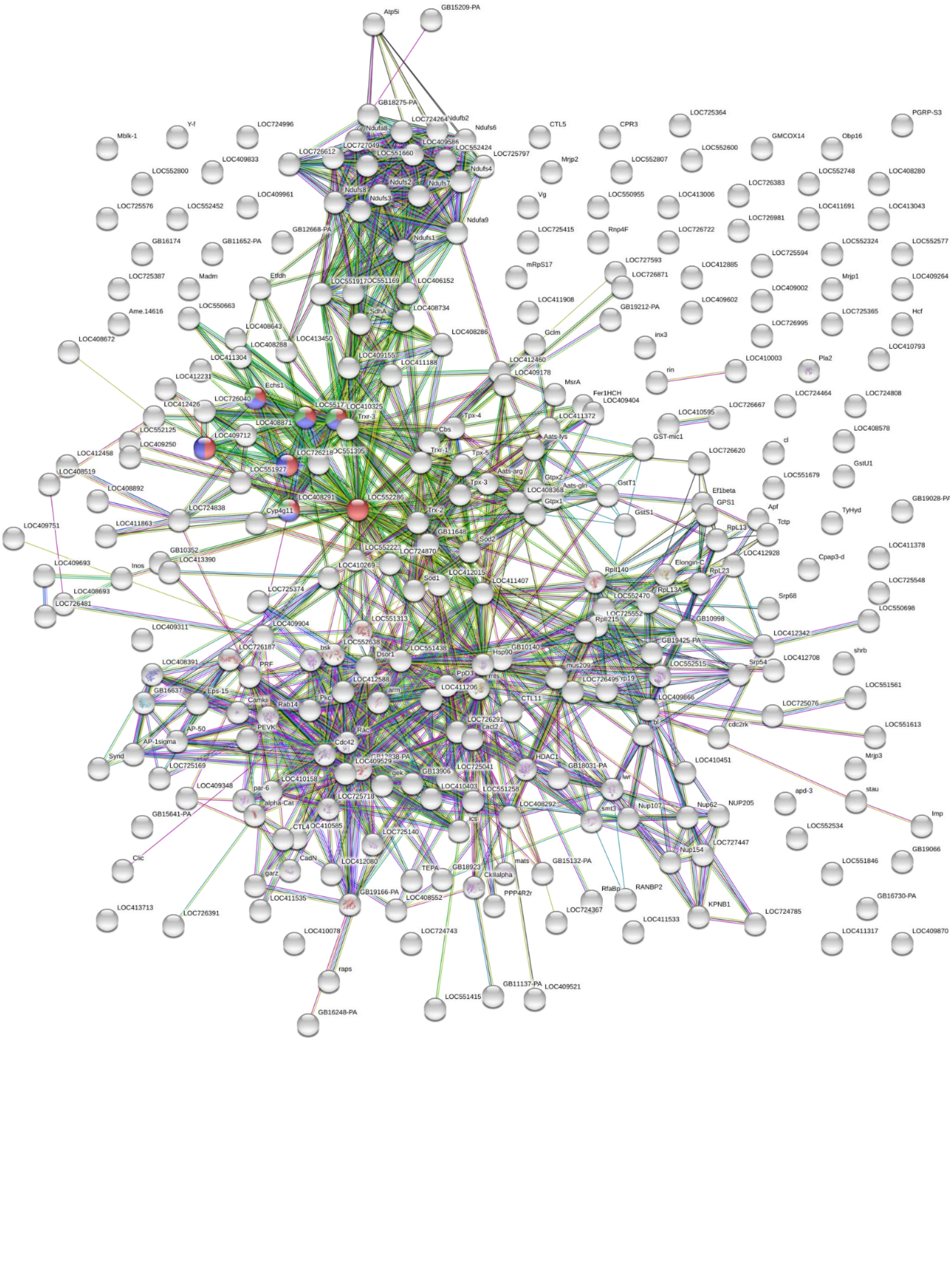

Figure S6. STRING pathway proteins: glutathione metabolism.

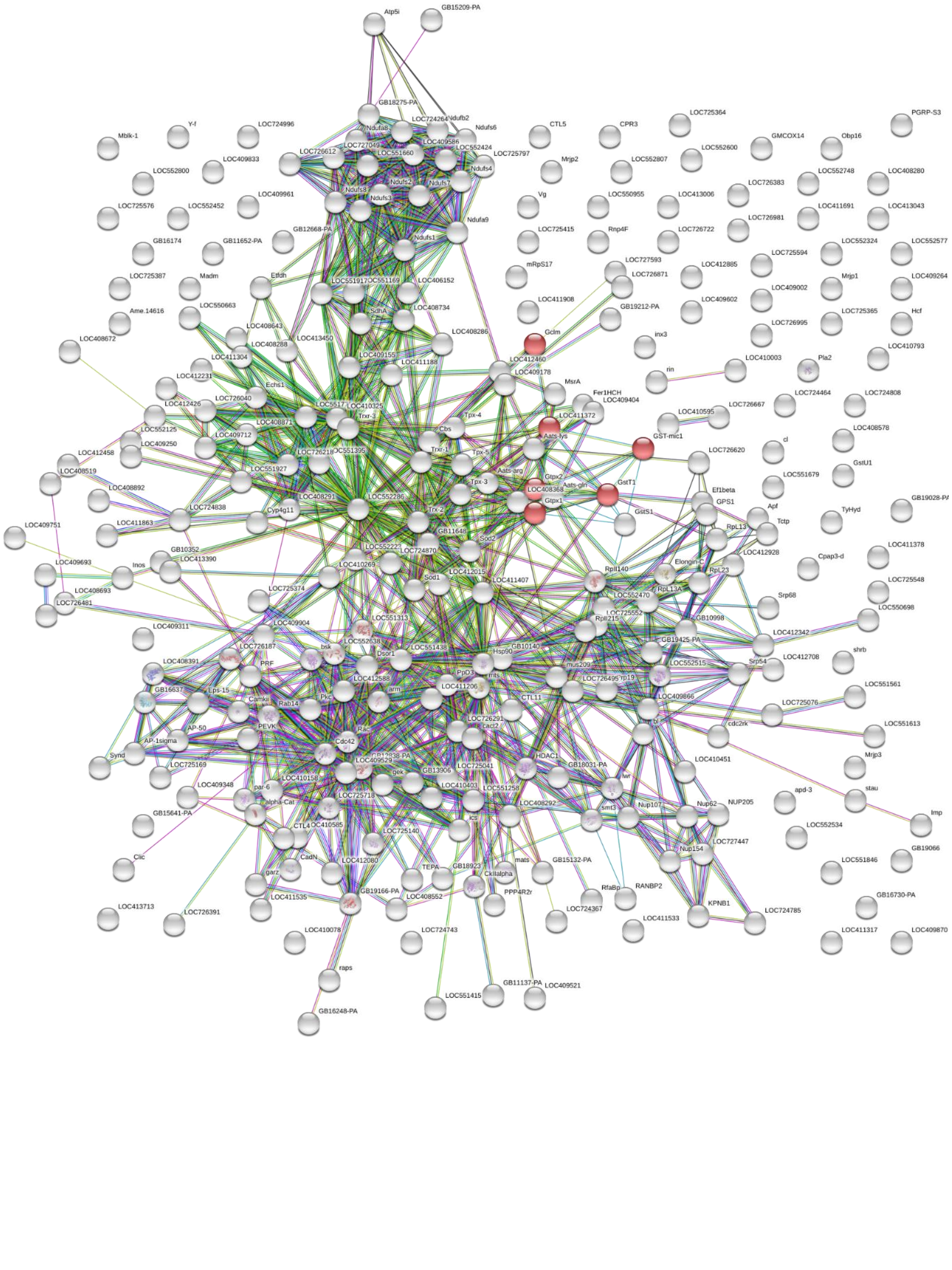

Figure S7. STRING pathway proteins: Wnt (red) and Hippo (blue) signaling pathways.

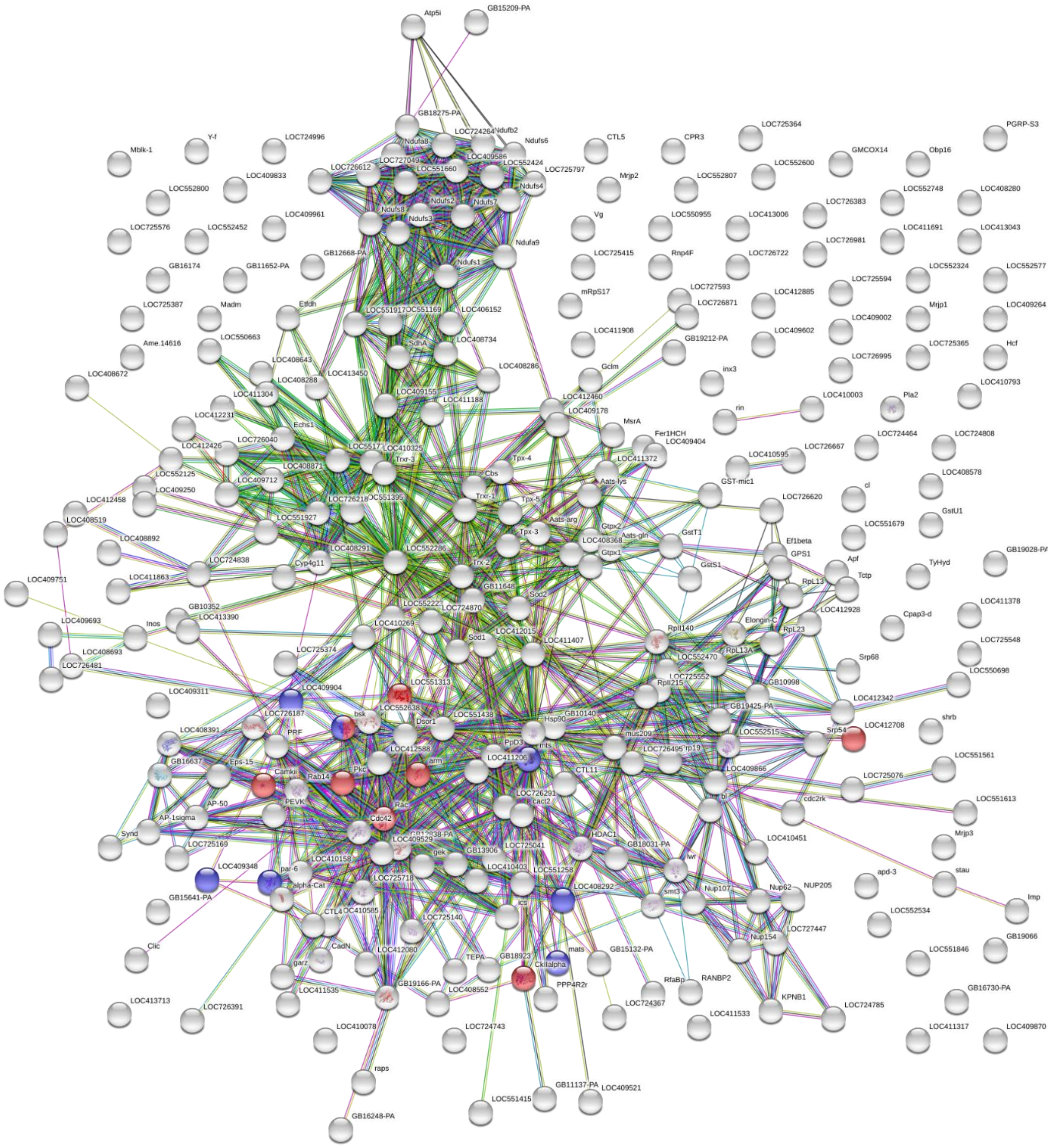

Figure S8. STRING pathway proteins: RNA transport.

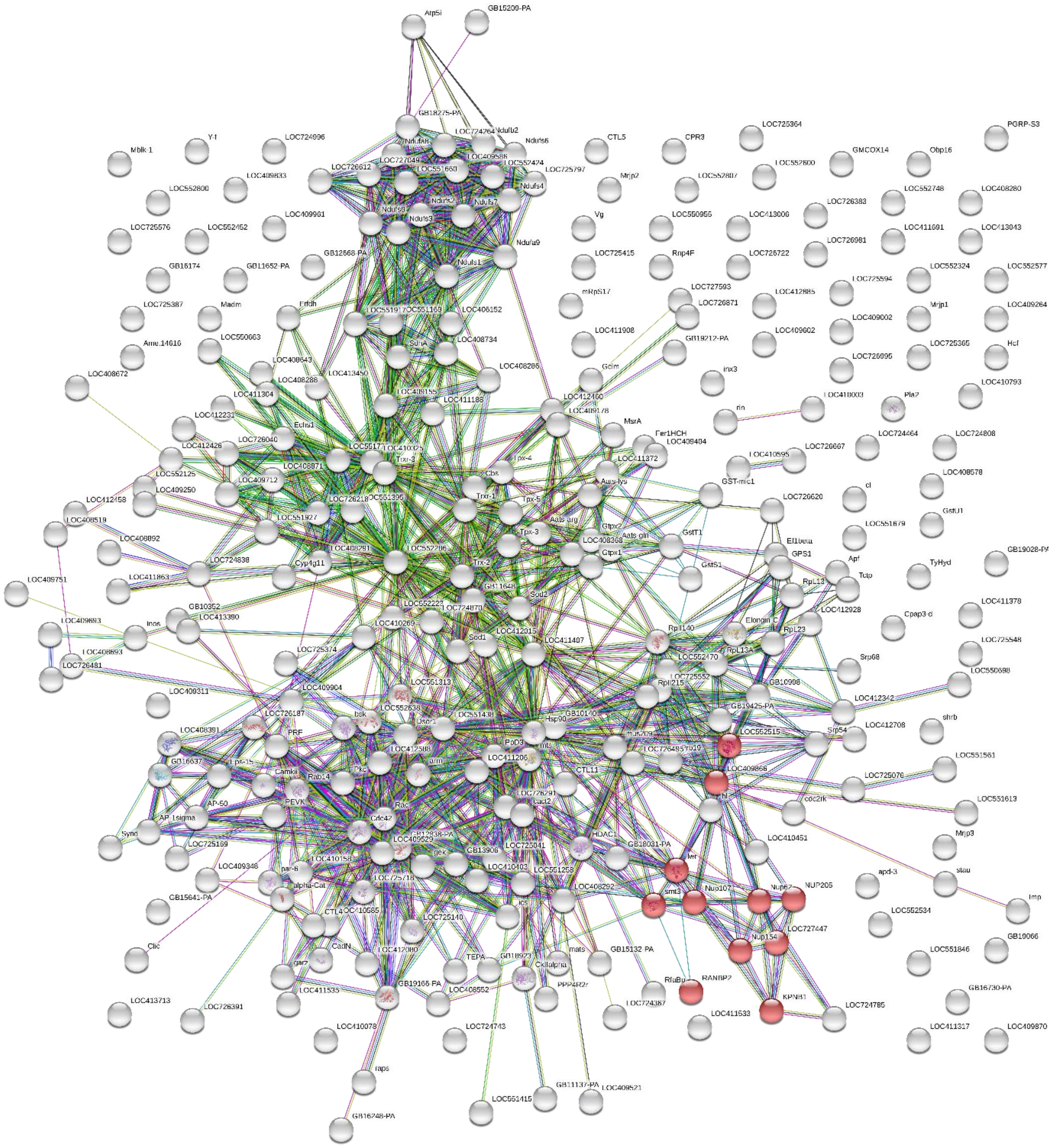

Figure S9. STRING pathway proteins: arachidonic acid metabolism.

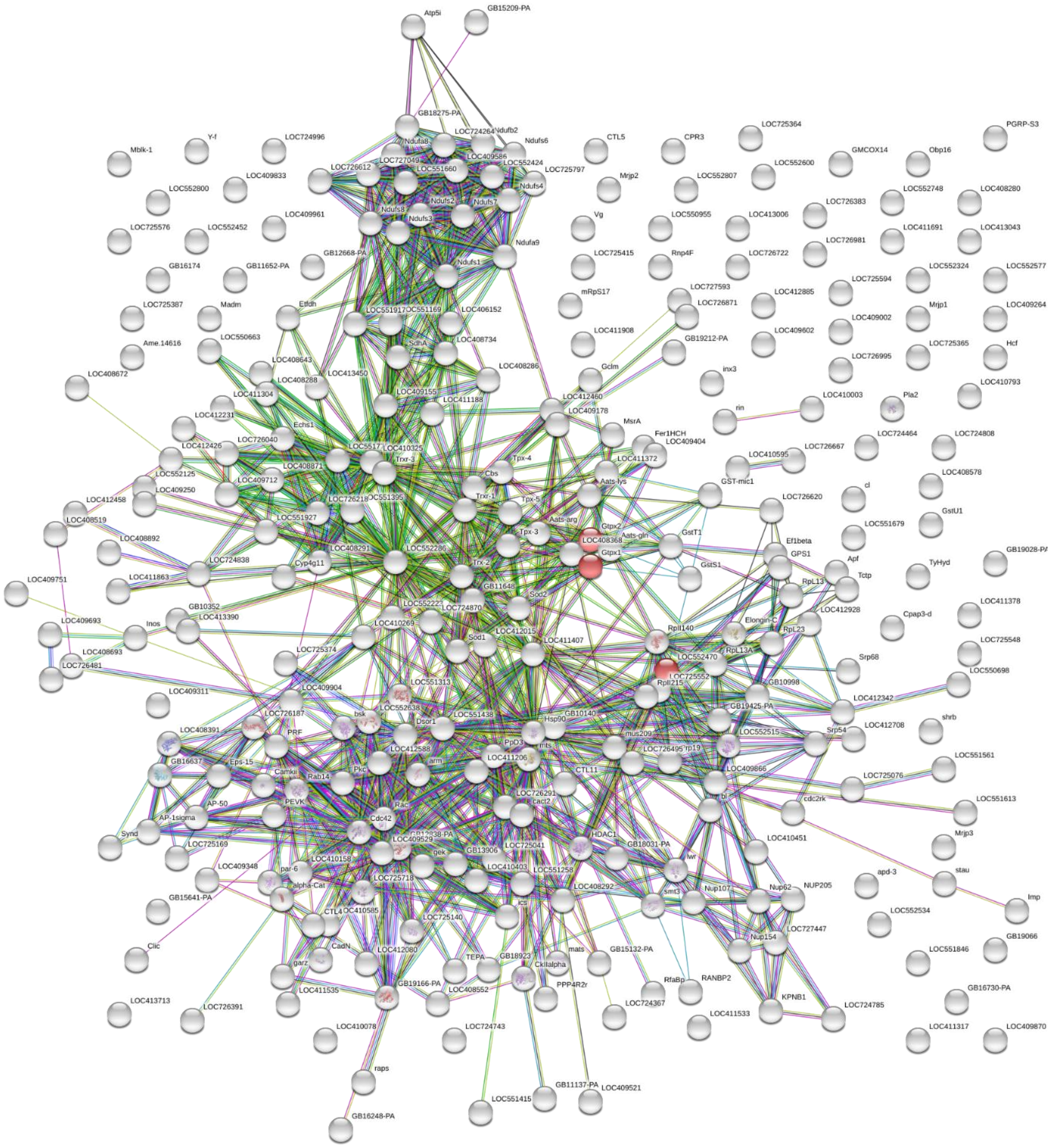

Figure S10. Thioredoxin-like fold INTERPRO domain.

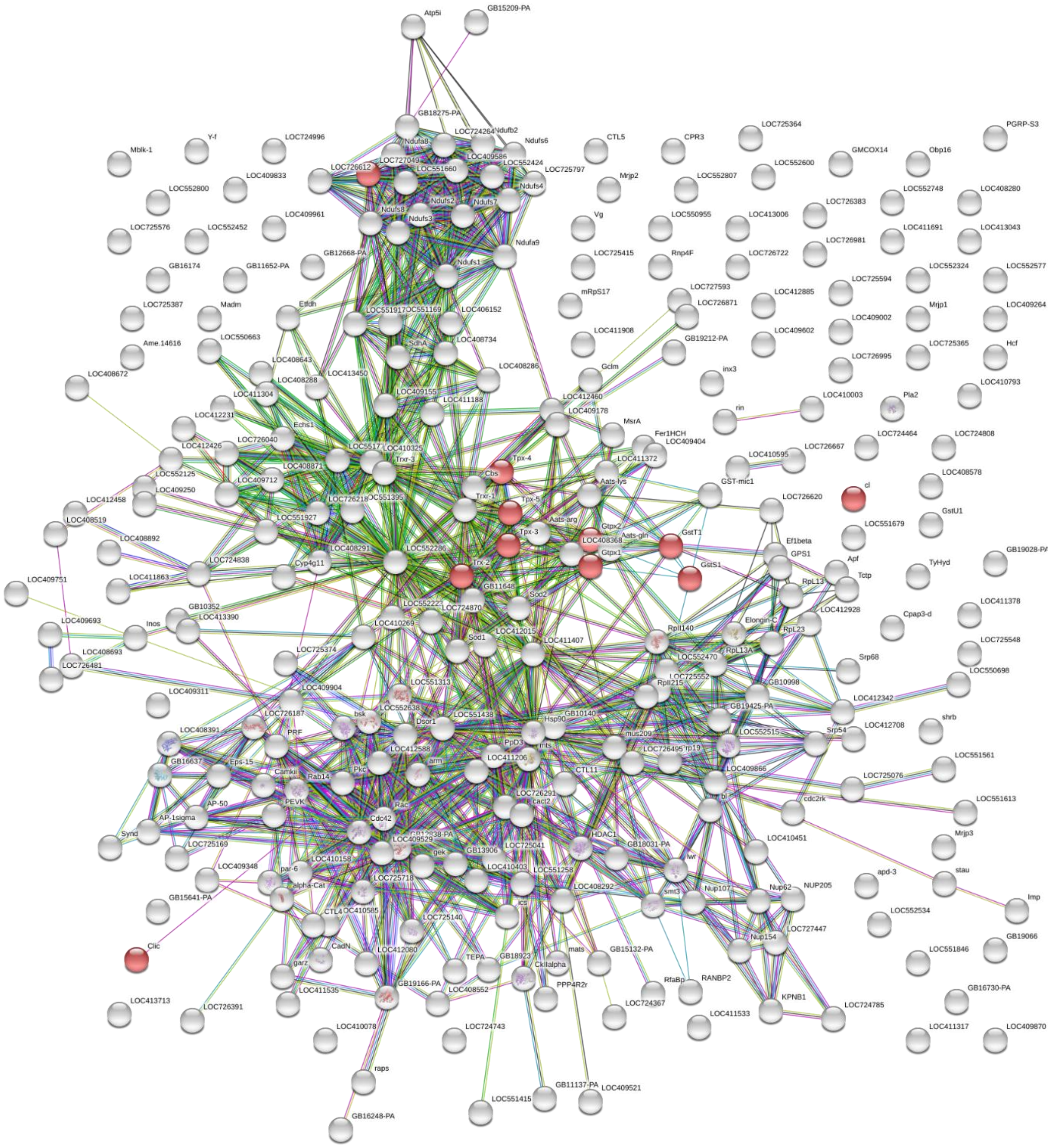

Search for proteins that are influenced by TGF-β

We successfully identified unique homologue proteins with analogous changes to those described previously by Xie *et al*.[26]. These results strongly support our hypothesis that *Varroa* secretions influence the TGF-β receptor. Thus, our study suggests that *Varroa* saliva contains TGF-β or its agonist as one of the main compounds influencing wound healing and vascularization and supports the influence on related proteins influenced by *Varroa* alone and together with *DWV*.

Table S4. Proteins that correspond to those that were influenced by TGF-β in study by Xie *et al*.[26]. According to Blastp similarity and same function, 51 proteins in our study corresponded to the reference study.

Protein (*Apis mellifera*)	Protein	TGF-β	VAR		Blastp Positives		DWV		DWV_VAR	
	**Signal Transduction**									
XP_016768828.1 PREDICTED: serine/threonine-protein kinase OSR1-like isoform X3 [*Apis mellifera*];>XP_006559526.1 PREDICTED: serine/threonine-protein kinase OSR1-like isoform X3 [*Apis mellifera*];>XP_006559524.1	Serine/threonine kinase 25	+	+	1	62%		-	0	++	1
XP_016770171.1 PREDICTED: LOW QUALITY PROTEIN: integrin beta-PS [*Apis mellifera*]	Integrin β4	+	+	1	52%		+	1	++	1
>XP_016771100.1 PREDICTED: integrin alpha-PS1 isoform X4 [*Apis mellifera*];>XP_016771099.1 PREDICTED: integrin alpha-PS1 isoform X3 [*Apis mellifera*];>XP_006565328.1 PREDICTED: integrin alpha-PS1 isoform X2 [*Apis mellifera*];>XP_625120.3 PREDICTED: integrin a	Integrin α3A	+	+	1	60%		+	1	+	1
>XP_016770171.1 PREDICTED: LOW QUALITY PROTEIN: integrin beta-PS [*Apis mellifera*]	Integrin β1	+	+	1	55%		+	1	++	1
>XP_006564353.1 PREDICTED: integrin-linked protein kinase homolog pat-4 [*Apis mellifera*]	Integrin-linked kinase	+	+	1	76%		+	1	+	1
>XP_016770171.1 PREDICTED: LOW QUALITY PROTEIN: integrin beta-PS [*Apis mellifera*]	Integrin β5	+	+	1	53%		+	1	++	1
>XP_006561448.1 PREDICTED: tyrosine-protein phosphatase 69D [*Apis mellifera*]	Tyrosine phosphatase PTPT9	+	+	1	57%		0	0	+	1
	**Intracellular components**									
>XP_016770377.1 PREDICTED: alpha-actinin, sarcomeric [*Apis mellifera*]	α-actinin	+	+	1	89%		+	1	+	1
>XP_016769972.1 PREDICTED: tropomyosin-2 isoform X24 [*Apis mellifera*];>XP_006571112.1 PREDICTED: tropomyosin alpha-3 chain isoform X8 [*Apis mellifera*];>XP_006571106.1 PREDICTED: tropomyosin alpha-3 chain isoform X6 [*Apis mellifera*]	β-tropomyosin 2	+	+	1	76%		-	0	+	1
>XP_625229.1 PREDICTED: catenin alpha isoform X4 [*Apis mellifera*];>XP_006563449.1 PREDICTED: catenin alpha isoform X3 [*Apis mellifera*];>XP_006563448.1 PREDICTED: catenin alpha isoform X2 [*Apis mellifera*];>XP_016772334.1 PREDICTED: catenin alpha isoform X1	Catenin src	+	+	1	75%		+	1	+	1
	**Extracellular components**									
>XP_392097.4 PREDICTED: collagen alpha-1(IV) chain [*Apis mellifera*]	α1 type IV collagen	+	+	1	50%		+	1	+	1
	**Nucleic acid binding**									
>XP_006562484.1 PREDICTED: eukaryotic translation initiation factor 4 gamma 2 isoform X2 [*Apis mellifera*];>XP_006562483.1 PREDICTED: eukaryotic translation initiation factor 4 gamma 2 isoform X2 [*Apis mellifera*];>XP_006562482.1 PREDICTED: eukaryotic transl	Translation initiation factor 2	+	+	1	59%		+	1	+	1
	**Cell cycle regulator**									
>XP_392973.4 PREDICTED: cyclin-dependent kinase 10 [*Apis mellifera*]	CDK9	+	+	1	59%	*	++	1	++	1
	**Cancer related proteins**									
>XP_006568906.1 PREDICTED: ubiquitin-conjugating enzyme E2 R2 isoform X2 [*Apis mellifera*];>XP_394314.2 PREDICTED: ubiquitin-conjugating enzyme E2 R2 isoform X1 [*Apis mellifera*]	E2 oncogene	+	+	1	82%		0	0	+	1
	**Transport**									
>XP_006557791.1 PREDICTED: solute carrier family 2, facilitated glucose transporter member 1-like isoform X9 [*Apis mellifera*];>XP_006557790.1 PREDICTED: solute carrier family 2, facilitated glucose transporter member 1-like isoform X8 [*Apis mellifera*];>XP_	Glucose transport protein	+	+	1	60%		+	1	+	1
	**Ubiquitins and elongation factors**									
>XP_392691.2 PREDICTED: translation elongation factor 2 isoform X2 [*Apis mellifera*];>XP_016767773.1 PREDICTED: translation elongation factor 2 isoform X1 [*Apis mellifera*]	Elongation factor 2	+	+	1	87%		+	1	+	1
>XP_016773030.1 PREDICTED: ubiquitin-conjugating enzyme E2 G2 isoform X3 [*Apis mellifera*]	Ubiquitin conjugating enzyme	+	+	1	28%		+	1	0	0
	**Enzyme**									
>XP_016772844.1 PREDICTED: glutathione S-transferase S1 isoform X1 [*Apis mellifera*]	Glutathione S-transferase	-	+	0	50%		+	1	+	1
>XP_006561429.1 PREDICTED: tyrosine-protein phosphatase corkscrew isoform X2 [*Apis mellifera*];>XP_006561428.1 PREDICTED: tyrosine-protein phosphatase corkscrew isoform X2 [*Apis mellifera*];>XP_003249854.1 PREDICTED: tyrosine-protein phosphatase corkscrew is	Protein tyrosine phosphatase	+	+	1	53%		-	0	+	1

>XP_624353.1 PREDICTED: aldose reductase-like [*Apis mellifera*]	Aldose reductase	+	+	1	74%		+	1	+	1
>XP_016770060.1 PREDICTED: protein disulfide-isomerase [*Apis mellifera*]	Disulfide isomerase	+	+	1	73%		+	1	+	1
>XP_006570475.1 PREDICTED: casein kinase II subunit alpha isoform X2 [*Apis mellifera*];>XP_623397.2 PREDICTED: casein kinase II subunit alpha isoform X1 [*Apis mellifera*];>XP_006570473.1 PREDICTED: casein kinase II subunit alpha isoform X1 [*Apis mellifera*];>	Casein kinase II	+	++	1	92%		++	1	++	1
>XP_006569964.1 PREDICTED: DNA-directed RNA polymerase III subunit RPC1 [*Apis mellifera*]	RNA polymerase II	+	++	1	57%		++	1	++	1
	**Cellular communication**									
>XP_392973.4 PREDICTED: cyclin-dependent kinase 10 [*Apis mellifera*]	cdc2	+	+	1	63%		++	1	++	1
>XP_625229.1 PREDICTED: catenin alpha isoform X4 [*Apis mellifera*];>XP_006563449.1 PREDICTED: catenin alpha isoform X3 [*Apis mellifera*];>XP_006563448.1 PREDICTED: catenin alpha isoform X2 [*Apis mellifera*];>XP_016772334.1 PREDICTED: catenin alpha isoform X1	Catenin α1	+	+	1	79%		+	1	+	1
	**Signal transduction**									
>XP_623996.3 PREDICTED: ras GTPase-activating protein-binding protein 2 [*Apis mellifera*]	Ras-GTPase-activating SH3 binding	+	+	1	74%		+	1	++	1
	**Intracellular components**									
>XP_624172.1 PREDICTED: probable actin-related protein 2/3 complex subunit 2 [*Apis mellifera*]	Actin-related protein 2/3 complex	+	+	1	84%		+	1	+	1
>XP_006561012.1 PREDICTED: myosin-IB [*Apis mellifera*]	Unconventional myosin-15	+	+	1	57%		+	1	+	1
>XP_006571121.1 PREDICTED: tropomyosin isoform X15 [*Apis mellifera*]	Nonmuscle tropomyosin 5	+	++	1	72%	* (ratus)	+	1	+	1
>XP_623834.1 PREDICTED: actin-related protein 1 [*Apis mellifera*]	Actin-related protein 1	+	++	1	94%		+	1	++	1
>XP_016769972.1 PREDICTED: tropomyosin-2 isoform X24 [*Apis mellifera*];>XP_006571112.1 PREDICTED: tropomyosin alpha-3 chain isoform X8 [*Apis mellifera*];>XP_006571106.1 PREDICTED: tropomyosin alpha-3 chain isoform X6 [*Apis mellifera*]	Tropomyosin isoform 2	+	+	1	76%		0	0	+	1
>NP_001153877.1 calpain-B [*Apis mellifera*]	Calpain	+	+	1	60%		+	1	+	1
>XP_625016.2 PREDICTED: myosin regulatory light chain sqh-like [*Apis mellifera*];>XP_623372.1 PREDICTED: myosin regulatory light chain sqh [*Apis mellifera*]	Myosin light chain 2	+	+	1	71%		+	1	++	1
	**Extracellular components**									
>XP_392097.4 PREDICTED: collagen alpha-1(IV) chain [*Apis mellifera*]	α-1(XVIII) collagen	+	+	1	40%	*	+	1	++	1
>XP_006567927.1 PREDICTED: DNA damage-binding protein 1 [*Apis mellifera*]	Damage-specific DNA binding	+	+	1	81%		+	1	+	1
	**Cell cycle regulator**									
>XP_396161.2 PREDICTED: calcyclin-binding protein [*Apis mellifera*]	Calcyclin binding protein	+	+	1	61%		0	0	++	1
	**Transport**									
>XP_625098.1 PREDICTED: V-type proton ATPase subunit E [*Apis mellifera*]	ATPase, H^+ transporting	+	+	1	73%		+	1	+	1
>XP_006560591.1 PREDICTED: calcium/calmodulin-dependent protein kinase II isoform X28 [*Apis mellifera*];>XP_006560589.1 PREDICTED: calcium/calmodulin-dependent protein kinase II isoform X26 [*Apis mellifera*];>XP_016767055.1 PREDICTED: calcium/calmodulin-depe	Ca^{2+}/calmodulin-dependent kinase	+	+	1	88%		+	1	+	1
	**Microtubular dynamics**									
>XP_016769399.1 PREDICTED: kinesin light chain isoform X6 [*Apis mellifera*];>XP_006567434.1 PREDICTED: kinesin light chain isoform X6 [*Apis mellifera*];>XP_006567433.1 PREDICTED: kinesin light chain isoform X5 [*Apis mellifera*];>XP_006567432.1 PREDICTED: kine	Kinesin light chain 1	+	+	1	80%		0	0	+	1
	**Ubiquitin and elongation factors**									
>XP_624702.2 PREDICTED: ubiquitin carboxyl-terminal hydrolase 5 [*Apis mellifera*]	Ubiquitin-specific protease 5	+	+	1	68%		+	1	++	1
>XP_006569957.1 PREDICTED: elongation factor 1-alpha isoform X1 [*Apis mellifera*];>XP_006569955.1 PREDICTED: elongation factor 1-alpha isoform X1 [*Apis mellifera*];>XP_006569954.1 PREDICTED: elongation factor 1-alpha isoform X1 [*Apis mellifera*];>XP_006569953	Elongation factor 1-α	+	+	1	91%		+	1	+	1
	**Enzyme**									
>XP_396126.2 PREDICTED: probable phosphoserine aminotransferase [*Apis mellifera*]	Phosphoserine aminotransferase	+	+	1	75%		+	1	++	1
>CAA34681.1 phospholipase A-2, partial [*Apis mellifera*];>P00630.3 RecName: Full=Phospholipase A2;Short=bvPLA2;AltName: Full=Allergen Api m I;AltName: Full=Phosphatidylcholine 2-acylhydrolase;AltName: Allergen=Api m 1;Flags: Precursor;>NP_001011614.1 p	Phospholipase A_2	+	+	1	60%		+	1	--	0
>XP_016767900.1 PREDICTED: proteasome subunit alpha type-5 [*Apis mellifera*]	Proteasome	+	+	1	88%		+	1	++	1
>XP_016767657.1 PREDICTED: sodium/potassium-transporting ATPase subunit alpha isoform X7 [*Apis mellifera*];>XP_016767656.1 PREDICTED: sodium/potassium-transporting ATPase subunit alpha isoform X6 [*Apis mellifera*];>XP_016767655.1 PREDICTED: sodium/potassium-	Na,K-ATPase α	+	+	1	77%		+	1	+	1
>XP_623084.1 PREDICTED: aldehyde dehydrogenase, mitochondrial [*Apis mellifera*]	Aldehyde dehydrogenase	+	+	1	79%		+	1	+	1
>XP_623105.1 PREDICTED: serine/threonine-protein phosphatase 2A catalytic subunit alpha isoform [*Apis mellifera*];>XP_019876139.1 PREDICTED: serine/threonine-protein phosphatase 2A catalytic subunit beta isoform [*Aethina tumida*]	Protein phosphatase 2A	+	+	1	98%		+	1	+	1
>XP_623198.1 PREDICTED: coatomer subunit alpha [*Apis mellifera*]	Coatomer protein	+	+	1	84%		+	1	++	1
>XP_006562044.2 PREDICTED: LOW QUALITY PROTEIN: dual specificity mitogen-activated protein kinase kinase 3-like [*Apis mellifera*]	MAP kinase kinase	+	+	1	74%		+	1	+	1
>XP_392943.3 PREDICTED: serine/threonine-protein phosphatase alpha-2 isoform [*Apis mellifera*];>XP_019878038.1 PREDICTED: serine/threonine-protein phosphatase alpha-2 isoform [*Aethina tumida*]	Protein phosphatase 1	+	+	1	94%		+	1	+	1
>XP_006569920.1 PREDICTED: sodium/potassium-transporting ATPase subunit beta-2 [*Apis mellifera*]	Na,K-ATPase β	+	+	1	51%		+	1	+	1
				**50**	**51**			**43**		**49**

Supplementary References

(as they are numbered in the main text)

26. Xie, L. *et al.* Transforming growth factor beta-regulated gene expression in a mouse mammary gland epithelial cell line. *Breast Cancer Res.* **5,** R187–R198 (2003).

176. Oksanen, J. *et al.* vegan: Community Ecology Package. *CRAN - The Comprehensive R Archive Network*, http://CRAN.R-project.org/package=vegan (2016).

178. Szklarczyk,D. *et al.* The STRING database in 2017: quality-controlled protein–protein association networks, made broadly accessible. *Nucleic Acids Res.* **45,** D362–D368 (2017).
